# Glasgow Early Treatment Arm Favirpiravir (GETAFIX) for adults with early stage COVID-19: A structured summary of a study protocol for a randomised controlled trial

**DOI:** 10.1186/s13063-020-04891-1

**Published:** 2020-11-19

**Authors:** Catherine R. Hanna, Kevin G. Blyth, Glenn Burley, Samantha Carmichael, Carol Evans, Samantha Hinsley, Ibrahim Khadra, Saye Khoo, Liz-Anne Lewsley, Robert R. Jones, Raman Sharma, Andrea Taladriz-Sender, Emma C. Thomson, Janet T. Scott

**Affiliations:** 1 CRUK Clinical Trials Unit, University of Glasgow, Glasgow, UK; 2 NHS Greater Glasgow & Clyde, Glasgow, UK; 3 Institute of Cancer Sciences, University of Glasgow, Glasgow, UK; 4 University of Strathclyde, Glasgow, UK; 5 Department of Pharmacology, University of Liverpool, Liverpool, UK; 6 Department of Tropical Disease Biology, Liverpool School of Tropical Medicine, Liverpool, UK; 7 MRC-University of Glasgow Centre for Virus Research, Institute of Infection, Immunity and Inflammation, University of Glasgow, Glasgow, UK

**Keywords:** COVID-19, Randomised controlled trial, protocol, favipiravir, inpatient, outpatient, early, pharmacokinetics, safety, tolerability, antiviral

## Abstract

**Objectives:**

The GETAFIX trial will test the hypothesis that favipiravir is a more effective treatment for COVID-19 infection in patients who have early stage disease, compared to current standard of care. This study will also provide an important opportunity to investigate the safety and tolerability of favipiravir, the pharmacokinetic and pharmacodynamic profile of this drug and mechanisms of resistance in the context of COVID-19 infection, as well as the effect of favipiravir on hospitalisation duration and the post COVID-19 health and psycho-social wellbeing of patients recruited to the study.

**Trial design:**

GETAFIX is an open label, parallel group, two arm phase II/III randomised trial with 1:1 treatment allocation ratio. Patients will be randomised to one of two arms and the primary endpoint will assess the superiority of favipiravir plus standard treatment compared to standard treatment alone.

**Participants:**

This trial will recruit adult patients with confirmed positive valid COVID-19 test, who are not pregnant or breastfeeding and have no prior major co-morbidities. This is a multi-centre trial, patients will be recruited from in-patients and outpatients from three Glasgow hospitals: Royal Alexandra Hospital; Queen Elizabeth University Hospital; and the Glasgow Royal Infirmary. Patients must meet all of the following criteria:

1. Age 16 or over at time of consent

2. Exhibiting symptoms associated with COVID-19

3. Positive for SARS-CoV-2 on valid COVID-19 test

4. Point 1, 2, 3, or 4 on the WHO COVID-19 ordinal severity scale at time of randomisation. (Asymptomatic with positive valid COVID-19 test, Symptomatic Independent, Symptomatic assistance needed, Hospitalized, with no oxygen therapy)

5. Have >=10% risk of death should they be admitted to hospital as defined by the ISARIC4C risk index: https://isaric4c.net/risk

6. Able to provide written informed consent

7. Negative pregnancy test (women of childbearing potential*)

8. Able to swallow oral medication

Patients will be excluded from the trial if they meet any of the following criteria:

1. Renal impairment requiring, or likely to require, dialysis or haemofiltration

2. Pregnant or breastfeeding

3. Of child bearing potential (women), or with female partners of child bearing potential (men) who do not agree to use adequate contraceptive measures for the duration of the study and for 3 months after the completion of study treatment

4. History of hereditary xanthinuria

5. Other patients judged unsuitable by the Principal Investigator or sub-Investigator

6. Known hypersensitivity to favipiravir, its metabolites or any excipients

7. Severe co-morbidities including: patients with severe hepatic impairment, defined as:

• greater than Child-Pugh grade A

• AST or ALT > 5 x ULN

• AST or ALT >3 x ULN and Total Bilirubin > 2xULN

8. More than 96 hours since first positive COVID-19 test sample was taken

9. Unable to discontinue contra-indicated concomitant medications

This is a multi-centre trial, patients will be recruited from in-patients and outpatients from three Glasgow hospitals: Royal Alexandra Hospital; Queen Elizabeth University Hospital; and the Glasgow Royal Infirmary.

**Intervention and comparator:**

Patients randomised to the experimental arm of GETAFIX will receive standard treatment for COVID-19 at the discretion of the treating clinician plus favipiravir. These patients will receive a loading dose of favipiravir on day 1 of 3600mg (1800mg 12 hours apart). On days 2-10, patients in the experimental arm will receive a maintenance dose of favipiravir of 800mg 12 hours apart (total of 18 doses). Patients randomised to the control arm of the GETAFIX trial will receive standard treatment for COVID-19 at the discretion of the treating clinician.

**Main outcomes:**

The primary outcome being assessed in the GETAFIX trial is the efficacy of favipiravir in addition to standard treatment in patients with COVID-19 in reducing the severity of disease compared to standard treatment alone. Disease severity will be assessed using WHO COVID 10 point ordinal severity scale at day 15 +/- 48 hours. All randomised participants will be followed up until death or 60 days post-randomisation (whichever is sooner).

**Randomisation:**

Patients will be randomised 1:1 to the experimental versus control arm using computer generated random sequence allocation. A minimisation algorithm incorporating a random component will be used to allocate patients. The factors used in the minimisation will be: site, age (16-50/51-70/71+), history of hypertension or currently obsess (BMI>30 or obesity clinically evident; yes/no), 7 days duration of symptoms (yes/no/unknown), sex (male/female), WHO COVID-19 ordinal severity score at baseline (1/2or 3/4).

**Blinding (masking):**

No blinding will be used in the GETAFIX trial. Both participants and those assessing outcomes will be aware of treatment allocation.

**Numbers to be randomised (sample size):**

In total, 302 patients will be randomised to the GETAFIX trial: 151 to the control arm and 151 to the experimental arm.

There will be an optional consent form for patients who may want to contribute to more frequent PK and PD sampling. The maximum number of patients who will undergo this testing will be sixteen, eight males and eight females. This option will be offered to all patients who are being treated in hospital at the time of taking informed consent, however only patients in the experimental arm of the trial will be able to undergo this testing.

**Trial Status:**

The current GETAFIX protocol is version 4.0 12^th^ September 2020. GETAFIX opened to recruitment on 26^th^ October 2020 and will recruit patients over a period of approximately six months.

**Trial registration:**

GETAFIX was registered on the European Union Drug Regulating Authorities Clinical Trials (EudraCT) Database on 15^th^ April 2020; Reference number 2020-001904-41 (https://www.clinicaltrialsregister.eu/ctr-search/trial/2020-001904-41/GB). GETAFIX was registered on ISRCTN on 7^th^ September 2020; Reference number ISRCTN31062548 (https://www.isrctn.com/ISRCTN31062548).

**Full protocol:**

The full protocol is attached as an additional file, accessible from the Trials website (Additional file 1). In the interest in expediting dissemination of this material, the familiar formatting has been eliminated; this Letter serves as a summary of the key elements of the full protocol. The study protocol has been reported in accordance with the Standard Protocol Items: Recommendations for Clinical Interventional Trials (SPIRIT) guidelines (see Additional file 2).

**Supplementary Information:**

**Supplementary information** accompanies this paper at 10.1186/s13063-020-04891-1.

## Supplementary Information

**Additional file 1.** Full study protocol

**Additional file 2.**

## Data Availability

The CRUK Clinical Trials Unit (Glasgow), which is co-ordinating the trial, will collect and store trial data, with access provided only to authorised personnel. NHS Greater Glasgow & Clyde and the University of Glasgow are the sponsors for this study based in the United Kingdom, will be using information from patients and their medical records in order to undertake this study and will act as the data controller for this study. The Co-Sponsor will keep identifiable information for 15 years after the study has finished. As there is a worldwide need to develop new treatments for COVID-19, it is possible that the data from the GETAFIX trial may be combined with data from another study in order to get the most meaningful and useful results. All data (personal, clinical, economic and data coming from research on biological material) collected will be treated in compliance with the European and UK applicable laws to ensure confidentiality is maintained.

