# Supplementary material for: Glasgow Early Treatment Arm Favirpiravir (GETAFIX) for adults with early stage COVID-19: A structured summary of a study protocol for a randomised controlled trial
Source: Trials. 2020 Nov 19;21:935. doi: 10.1186/s13063-020-04891-1 (PMC7675389; doi:10.1186/s13063-020-04891-1)
Supplement: Supplementary file 1 — Additional file 1. Full study protocol [file 13063_2020_4891_MOESM1_ESM.pdf]

GETAFIX

Glasgow **E**arly **T**reatment **A**rm **Favipiravir**$^{\chi}$: A randomised controlled study of favipiravir as an early treatment arm in COVID-19 patients

Version 4.0 15/Sep/2020

EudraCT Ref:	2020-001904-41
ISRCTN No:	ISRCTN31062548
Protocol No:	GETAFIX-2020
IRAS No:	283151
Sponsor Ref:	GN20ID159
Sponsor/Co-Sponsor:	NHS Greater Glasgow and Clyde/University of Glasgow
Funder:	Chief Scientist Office
Grant Reference:	COV/GLA/20/03

Professor Rob Jones on behalf of:

CRUK Clinical Trials Unit, Glasgow (CRUK CTU)

(Partner of Cancer Clinical Trials Unit Scotland (CaCTUS))

Trial Management Group

Chief Investigator	**Professor Rob Jones**<br>Beatson Wet of Scotland Cancer Centre<br>Glasgow G12 0YN<br>Tel: 0141 301 7062<br>Email: r.jones@beatson.gla.ac.uk
Lead Collaborator	**Dr Janet Scott**<br>MRC-University of Glasgow Centre for Virus Research<br>janet.scott@glasgow.ac.uk
Co-Investigators	**Prof Kevin Blyth**<br>University of Glasgow Institute of Cancer Sciences<br>Kevin.Blyth@glasgow.ac.uk<br><br>**Prof Rob Jones**<br>Beatson West of Scotland Cancer Centre<br>r.jones@beatson.gla.ac.uk<br><br>**Prof Emma Thompson**<br>MRC-University of Glasgow Centre for Virus Research<br>Emma.Thomson@glasgow.ac.uk<br><br>**Prof Glenn Burley**<br>University of Strathclyde<br>glenn.burley@strath.ac.uk<br><br>**Dr Catherine Hanna**<br>Beatson West of Scotland Cancer Centre<br>Catherine.Hanna@glasgow.ac.uk<br><br>**Dr Ibrahim Khadra**<br>University of Strathclyde<br>Ibrahim.khadra@strath.ac.uk
Lead Statistician/Co-Investigator	**Samantha Hinsley**<br>CRUK Clinical Trials Unit<br>Beatson West of Scotland Cancer Centre<br>Samantha.Hinsley@glasgow.ac.uk
Project Manager	**Carol Evans/Liz-Anne Lewsley**<br>Cancer Research UK Clinical Trials Unit<br>Beatson West of Scotland Cancer Centre<br>Tel: 0141 301 7189/7193<br>Email: Carol.Evans@glasgow.ac.uk

	Liz-Anne.Lewsley@glasgow.ac.uk
Clinical Trial Co-ordinator	**Laura Divers**<br>Cancer Research UK Clinical Trials Unit<br>Beatson West of Scotland Cancer Centre<br>Tel: 0141 301 7246<br>Email: Laura.Divers@glasgow.ac.uk
Pharmacovigilance Manager	**Jacqueline Gourlay**<br>CRUK Clinical Trials Unit<br>Beatson West of Scotland Cancer Centre<br>Jacqueline.Gourlay@glasgow.ac.uk
Pharmacovigilance CTC	**Pamela Fergusson**<br>CRUK Clinical Trials Unit<br>Beatson West of Scotland Cancer Centre<br>Pamela.Fergusson@glasgow.ac.uk
Quality Assurance Manager	**Michaela Rodger**<br>CRUK Clinical Trials Unit<br>Beatson West of Scotland Cancer Centre<br>Michaela.Rodger@glasgow.ac.uk
Clinical Trial Monitor	**Stacey Chapman**<br>CRUK Clinical Trials Unit<br>Beatson West of Scotland Cancer Centre<br>Stacey.Chapman@glasgow.ac.uk
Sponsor Pharmacist	**Dr Samantha Carmichael**<br>Research and Development, NHS Greater Glasgow & Clyde<br>Ward 11, Dykebar Hospital<br>Paisley<br>Tel: 0141 314 0232<br>Samantha.Carmichael@ggc.scot.nhs.uk<br><br>**Dr Elizabeth Douglas**<br>Research and Development, NHS Greater Glasgow & Clyde<br>Ward 11, Dykebar Hospital<br>Paisley<br>Tel: 0141 314 4073<br>Elizabeth.Douglas@ggc.scot.nhs.uk
Co-Sponsor Representative (NHS GG&C)	**Joanne McGarry**<br>Research and Development, NHS Greater Glasgow and Clyde<br>Ward 11, Dykebar Hospital<br>Paisley<br>Tel: 0141 314 4001<br>Joanne.McGarry@ggc.scot.nhs.uk

Co-Sponsor Representative (UoG)	**Debra Stuart**<br>University of Glasgow<br>Glasgow<br>Tel: 0141 330 4539<br>debra.stuart@glasgow.ac.uk

Co-Sponsors

NHS Greater Glasgow & Clyde Representative

Joanne McGarry
Research Coordinator
NHS Greater Glasgow and Clyde
Clinical Research and Development Central Office
Ward 11, Dykebar Hospital
Grahamston Road
Paisley PA2 7DE
Tel: 0141 314 4001
Email: joanne.mcgarry@ggc.scot.nhs.uk

University of Glasgow Representative

Dr Debra Stuart
Glasgow University
Head of Research Governance
College of Medical, Veterinary and Life Sciences
University of Glasgow Room 327
Wolfson Medical School Building
University Avenue Glasgow G12 8QQ
0141 330 4539
Email: debra.stuart@glasgow.ac.uk

Funder(s):

Chief Scientist Office, Scotland

This trial will be performed according to the UK Policy Framework for Health and Social Care (2017) Medicines for Human Use (Clinical Trials) Regulations, 2004 SI 2004:1031 (as amended) and World Medical Association Declaration of Helsinki Ethical Principles for Medical Research Involving Human Subjects 1964 (as amended)

The study will comply with current government, MHRA and HRA guidance regarding COVID-19.

Protocol Authorisation

Name: Prof Rob Jones

Role: Chief Investigator

Date:

Signature:

Name: Samantha Hinsley

Role: Lead Statistician

Date:

Signature:

Name: Joanne McGarry

Role: Co-Sponsor Representative

Date:

Signature:

Trial Summary

Title

Glasgow Early Treatment Arm Favipiravir χ (GETAFIX): A randomised controlled study of favipiravir as an early treatment arm in COVID-19 patients

Background

Favipiravir is an antiviral agent developed by Toyama Chemical Co., Ltd. Favipiravir is approved in Japan for novel or re-emerging influenza virus infection in which other anti-influenza virus agents are not effective or insufficiently effective and the government decides to use the drug as a countermeasure against such influenza viruses. Its mechanism of action is selective inhibition of viral RNA polymerase by its triphosphorylated form (T-705RTP) and it may also be effective for RNA viruses other than influenza virus. It has been reported to be effective against Ebola virus,[1, 2] Arenaviridae, Lassa Fever, and Bunyaviridae both *in vitro* and *in vivo*.[1,2,3]

The new coronavirus (SARS-CoV-2), reported for the first time in China at the end of 2019 belongs to the Coronaviridae family of viruses within the order Nidovirales. Infection with this virus is associated with acute respiratory infection-like symptoms such as pyrexia, cough, chills, and pneumonia. As of 12th April 2020 there were 78 995 confirmed cases in the UK, and 9875 deaths since the beginning of 2020. Globally the number of confirmed cases stood at 1, 696 588 with 105 952 deaths.[3]

Wang *et al*. (2020) reported that the EC_{50} of favipiravir against SARS-CoV-2 virus in Vero E6 cells was 61.88µM.[4] This corresponds to a concentration of 9.72 µg/ml. By comparison, the EC50 for Ebola zaire was 10.5µg/mL [4]

In addition, when favipiravir administered with aerosolized interferon to 36 Chinese patients, shortened the viral clearance time from 11 days to 4, and showed significant improvement in chest imaging (91% vs. 62%) compared to Lopinavir/ritonavir Kaletra®, also administered with aerosolized interferon. Patients taking favipiravir also had fewer adverse events than those taking Lopinavir/ritonavir [5].

In a clinical pharmacology study in which 1800mg of favipiravir was administered twice daily on Day 1 and 800mg of favipiravir was administered twice daily on Day 2 and thereafter (1800/800mg twice

daily) for 22 days to healthy Japanese adult males aged 20 to 39 years, the peak plasma concentration of favipiravir remained at approximately 87 to 104µg/mL on Day 5 and after, and C_{min} remained at approximately 56 to 75µg/mL after the 2nd administration on Day 1. This value exceeds 9.72µg/mL, the EC_{50} of favipiravir for the SARS-CoV-2 virus mentioned above and is significant even when the human protein binding rate of favipiravir of approximately 50% is taken into consideration. Therefore, we propose that favipiravir administration may achieve exposures in humans that may be potentially efficacious in the treatment of COVID-19.

Considering that this viral infection has such a relatively high infectivity and that the outbreak is expected to continue, the development of effective treatments for COVID-19 are urgently needed. A reduction in time to viral clearance could help ameliorate the progression of the disease in severely affected patients and reduce transmission and hospital stay in milder cases.

Design

Open-label randomised phase II/III trial

Aims

Primary Aim: To evaluate the efficacy of favipiravir in patients with SARS-CoV-2 infection (COVID-19).

Table 1: Objectives and Endpoints

**Primary Objective**	**Primary Endpoint**	**Timepoint of assessment**
Assess the efficacy of favipiravir in addition to standard care in patients with COVID-19 in reducing the severity of disease compared to standard care alone	Clinical status as assessed by WHO COVID 10 point ordinal severity scale* at day 15	Day 15 (+/- 48 hours)
**Secondary objectives**	**Secondary Endpoints**	**Timepoint of assessment**
Assess the effect of favipiravir in addition to standard care in the study population compared to standard care alone	Proportion of patients meeting level 7 or above on the WHO COVID 10 point ordinal severity scale or dead by day 29<br><br>WHO COVID 10 point ordinal severity scale level	Up to and including day 29<br><br>Days 8, 29, 60

	Overall survival<br><br>***Inpatients only:***<br>Viral clearance on or before d8 **<br><br>***Inpatients only:***<br>Duration of pyrexia in days (defined as a temperature ≥ 38°C)	Up to and including day 60<br><br><br>Day 8<br><br><br>Up to and including day 60
Evaluate the safety and tolerability of favipiravir in the study population	Adverse events	Up to and including day 60
**Exploratory objectives**	**Exploratory endpoints**	**Timepoint of assessment**
Evaluate the effect of favipiravir on duration of hospitalisation	Duration of hospitalisation (days)	Up to and including day 60
Understand the pharmacokinetics of favipiravir	Evaluate the population pharmacokinetics of this dose of favipiravir in the study population and assess the relationship between the pharmacokinetics of favipiravir in the study population and viral load	Please see Appendix 5 for schedule of sampling
Understand the pharmacodynamics of favipiravir	Change in viral load with time<br><br>***Outpatients only***:<br>Viral clearance on or before d8 **	Up to and including day 60<br><br><br>Day 8
Understand mechanisms of resistance to favipiravir	Sequence of virus in cases of treatment failure	Up to and including day 60
Explore the immunological and biometric markers contributing to the clinical condition of patients in the study population	Evaluate the relationship between immunological and biometric markers and the clinical outcomes of the study	Up to and including day 60
Post COVID19 health and psycho-social consequences	Evaluate the relationship between post COVID19 health and psycho-social consequences and the clinical outcomes of the study	Up to and including day 60

*See Appendix 1 for WHO COVID-19 10 point ordinal severity scale.
** Defined as a negative SARS-CoV-2 PCR result

Population

Adult patients with confirmed positive valid COVID-19 test, who are not pregnant or breastfeeding and have no prior major co-morbidities.

Eligibility

Eligibility criteria:

Inclusion criteria

1. Age 16 or over at time of consent
2. Exhibiting symptoms associated with COVID-19
3. Positive for SARS-CoV-2 on valid COVID-19 test
4. Point 2, 3, or 4 on the WHO COVID-19 ordinal severity scale at time of randomisation. (Symptomatic Independent, Symptomatic assistance needed, Hospitalized, with no oxygen therapy)
5. Have >=10% risk of death should they be admitted to hospital as defined by the ISARIC4C risk index: https://isaric4c.net/risk
6. Able to provide written informed consent
7. Negative pregnancy test (women of childbearing potential*)
8. Able to swallow oral medication

Exclusion criteria

1. Renal impairment requiring, or likely to require, dialysis or haemofiltration
2. Pregnant or breastfeeding
3. Of child bearing potential* (women), or with female partners of child bearing potential (men) who do not agree to use adequate contraceptive measures for the duration of the study and for 3 months after the completion of study treatment
4. History of hereditary xanthinuria
5. Other patients judged unsuitable by the Principal Investigator or sub-Investigator
6. Known hypersensitivity to favipiravir, its metabolites or any excipients
7. Severe co-morbidities including: patients with severe hepatic impairment, defined as:
    - (greater than Child-Pugh grade A- see Appendix 4,
    - AST or ALT > 5 x ULN
    - AST or ALT >3 x ULN and Total Bilirubin > 2xULN
8. More than 96 hours since first positive COVID19 test sample was taken

9. Unable to discontinue contra-indicated concomitant medications (section 6.7)

* Non-childbearing potential must be evidenced by fulfilling one of the following criteria at screening:

- Post-menopausal defined as aged more than 50 years and amenorrhoeic for at least 12 months following cessation of all exogenous hormonal treatments.
- Prior irreversible surgical sterilisation by hysterectomy, bilateral oophorectomy or bilateral salpingectomy but not tubal ligation

Treatment

Patients will be randomised on a 1:1 basis to the following arms:

Control Arm: Standard Treatment for COVID-19 infection

OR

Experimental Arm: Standard Treatment for COVID-19 plus favipiravir:

- Day 1: Loading Dose favipiravir 1800mg (9 x 200mg tablets) 12 hours apart (2 doses)
- Days 2 -10 Maintenance Dose favipiravir 800mg (4 x 200mg tablets) every 12 hours (18 doses).

Duration

302 patients will be randomised and treated. All randomised participants will be followed up until death or 60 days post-randomisation (whichever is sooner).

CONTENTS

TRIAL MANAGEMENT GROUP.......... 2

CO-SPONSORS.......... 5

FUNDER(S): 5

PROTOCOL AUTHORISATION.......... 6

TRIAL SUMMARY.......... 7

1 INTRODUCTION & BACKGROUND.......... 24

1.1 IMP: Prior Experience in COVID-19 and dose selection.......... 25
1.2 Trial Rationale and Hypothesis.......... 27

2 TRIAL OBJECTIVES.......... 28

3 TRIAL DESIGN.......... 29

3.1 Trial population.......... 30
3.2 Eligibility criteria.......... 30
3.3 Identification of participants and consent.......... 31
3.4 Registration/Randomisation.......... 32
3.5 Withdrawal.......... 34
3.5.1 Withdrawal of Patients from Trial Treatment.......... 34
3.5.2 Withdrawal from Trial.......... 34

4 TRIAL PROCEDURES.......... 35

4.1 Trial Endpoints.......... 35
4.2 Trial Schedule.......... 35
4.2.1 Schedule for Inpatients.......... 35
4.2.2 Schedule for outpatients.......... 37
4.3 Laboratory Tests.......... 40
4.3.1 Routine blood tests.......... 40
4.3.2 Valid COVID19 Test for diagnosis and viral clearance.......... 40
4.3.3 Viral Load for pharmacodynamics.......... 40
4.3.4 Pharmacokinetic/Pharmacodynamic sampling.......... 40

5 TREATMENTS.......... 41

5.1 Treatment Arms.......... 41
5.2 Specific Drug Information.......... 42
5.2.1 Favipiravir.......... 42
5.3 Dispensing, Accountability and Administration.......... 42
5.3.1 Favipiravir Dispensing and Accountability.......... 42
5.3.2 Favipiravir Administration.......... 43
5.4 Trial Drug Supplies.......... 43
5.4.1 Favipiravir Supplies.......... 43
5.5 Trial Treatment Recording.......... 44
5.6 Concomitant Therapy.......... 44
5.7 Prohibited Therapy/Prohibited Medications/Permitted Concomitant Medications.......... 44

5.7.1 Interactions with other medications 45
5.7.2 Adverse Reactions 46
5.8 Dose Modifications for toxicity 46
5.8.1 Treatment Interruptions 47
5.8.2 Dose Modifications during Favipiravir Treatment 47
5.9 Participation in concurrent clinical trials 47
5.10 Duration of Trial Participation 47
5.10.1 Duration of Trial Treatment 48
5.10.2 Duration of Trial Follow-up 48

6 SAFETY REPORTING 49

6.1 Pharmacovigilance 49
6.1.1 Definitions 49
Serious Adverse Reaction (SAR) 50
Unexpected Serious Adverse Reaction (known as a SUSAR 50
6.1.2 Detecting, Recording and Reporting of Adverse Events 51
6.1.3 Assessment of Adverse Events 51
6.1.4 Reporting of a Serious Adverse Event 52
6.1.5 Identifying SUSARs 54
6.1.6 Reporting of a SUSAR 54
6.1.7 Other Unexpected Events Requiring Regulatory Reporting 55
6.2 Pregnancy 56
6.2.2 Medication Errors and Overdose 58
6.2.3 Development Safety Update Reports 59
6.2.4 Auxiliary Medicinal Products (AMPs) 59
6.2.5 Reference Safety Information 59
6.2.6 Changes to the RSI or Risk-Benefit Assessment 59
6.2.7 Requirements for DSUR Reporting and the Maintenance of RSI in the Follow-Up Period 60

7 STATISTICS AND DATA ANALYSIS 60

7.1 Trial Design and Sample Size 60
7.1.1 Analysis Plan 61
7.1.2 Primary Efficacy Analysis 61
7.1.3 Secondary Efficacy Analysis 62
7.1.4 Exploratory Endpoint Analysis 62
7.1.5 Safety 63
7.2 Interim Analysis 63

8 TRIAL CLOSURE 64

8.1 End of Trial Notification/Declaration of the End of a Study Form 65
8.2 Clinical Trial Summary Report 65
8.3 Temporary Halt of a trial 65
8.4 Early Termination of a Trial 65

9 DATA HANDLING 66

9.1 CRFs 66
9.2 Central Review of Data 66

9.3 Data Escalation Processes ... 67
9.4 Record Retention and archiving ... 67
9.5 PK/PD data analysis ... 68

10 TRIAL MANAGEMENT ... 68

10.1 Trial Start Up ... 68
10.2 Core Documents ... 69
10.3 Management of protocol deviations and violations ... 69
10.4 Trial Management Group (TMG) ... 70
10.5 COVID-19 Umbrella Trial Steering Committee (COVID-19 UTSC) ... 70
10.6 COVID-19 Umbrella Independent Data Monitoring Committee (COVID-19 UIDMC) ... 70

11 REGULATORY ISSUES ... 71

11.1 Clinical Trials Authorisation (CTA) ... 71
11.2 Ethics Approval ... 71
11.3 Consent ... 71
11.4 Confidentiality ... 72
11.5 Liability, Indemnity and Insurance ... 73
11.6 Sponsor(s) ... 73
11.7 Funding ... 73
11.8 Protocol Amendments ... 73
11.9 Allocation of Trial Responsibilities ... 74
11.9.2 Chief Investigator (CI) ... 74
11.9.3 CRUK Clinical Trials Unit (CTU) ... 74
11.9.4 Participating Site ... 75
11.9.5 Principal Investigator (PI) ... 75
11.10 Non-medical Prescribing (if appropriate) ... 75

12 QUALITY ASSURANCE ... 76

12.1 Audits and Inspections ... 76
12.2 On Site and Telephone Monitoring ... 76
12.3 Protocol non-compliance ... 76

13 PUBLICATION POLICY ... 77

14 REFERENCES ... 77

15 APPENDICES ... 78

Appendix 1 : WHO 10-point COVID Severity Scale ... 79
Appendix 2: World Health Organisation (WHO) performance score ... 81
Appendix 3: Questionnaire ... 82
Appendix 4 Child Pugh Classification ... 84
Appendix 5 PK Sampling Schedule ... 85

Abbreviations

AE	Adverse Event
AMP	Auxiliary Medicinal Product
AR	Adverse Reaction
ALT	Alanine Aminotransferase
AST	Aspartate Transaminase
CRUK	Cancer Research UK
CRP	C-reactive Protein
CTA	Clinical Trial Authorisation
CTA	Clinical Trial Agreement
CTC	Clinical Trial Coordinator
CTIMP	Clinical Trial of an Investigational Medicinal Product
CTU	Clinical Trials Unit
CI	Chief Investigator
CTIMP	Clinical Trial of an Investigational Medicinal Product
CTM	Clinical Trial Monitor
eCRF	Electronic Case Report Form
EU	European Union
GCP	Good Clinical Practice
GP	General Practitioner
IB	Investigator Brochure
ICF	Informed Consent Form
ICU	Intensive Care Unit
IMP	Investigational Medicinal Product
IND	Investigational New Drug
MHRA	Medicines and Healthcare Products Regulatory Agency
PCR	Polymerase chain reaction
PD	Pharmacodynamics
PK	Pharmacokinetics

PM	Project Manager
PNF	Pregnancy Notification Form
PV	Pharmacovigilance
R&D	Research and Development
REC	Research Ethics Committee
RNA	Ribonucleic Acid
RSI	Reference Safety Information
PI	Principal Investigator
PIS	Patient Information Sheet
REC	Research Ethics Committee
SAE	Serious Adverse Event
SAR	Serious Adverse Reaction
SARS	Severe Acute Respiratory Syndrome
SUSAR	Suspected Unexpected Serious Adverse Reaction
SOP	Standard Operating Procedures
TMG	Trial Management Group
ULN	Upper Limit of Normal
UTI	Urinary Tract Infection
UTSC	Umbrella Trials Steering Committee
UIDMC	Umbrella Independent Data Monitoring Committee
WHO	World Health Organisation

GETAFIX Trial Flow Chart

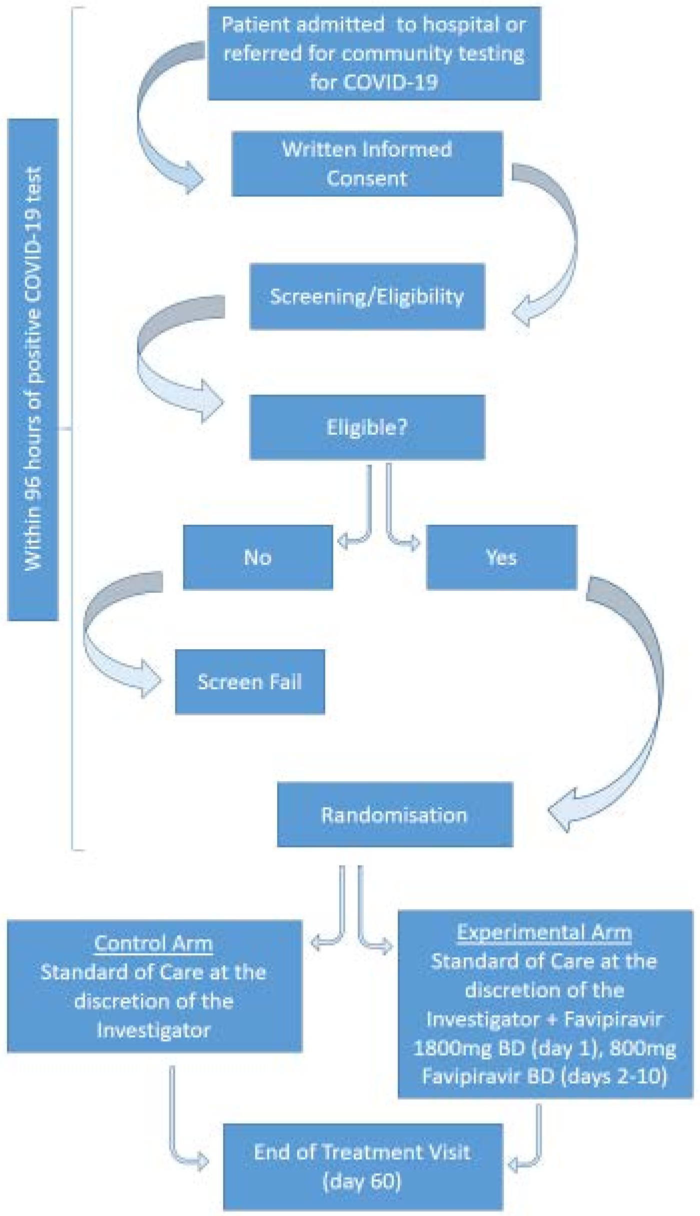

Schedule of Events for inpatients

Investigation	Screening (d-4 to day 1)	Day 1	Day 2	Day 3	Day 4	Day 5	Day 6	Day 7	Day 8	Day 9	Day 10	Day 15[5]	Follow-up(day 29)[6]	Follow-up (day 60)[6]
Written informed consent	X													
Demographic and Medical History	X													
Randomisation	X													
Observations[1]	X	X	X	X	X	X	X	X	X	X	X	X	X	X
Height and weight	X											X	X	X
Physical Exam	X	Symptom directed where clinically indicated												
AE Evaluation	Continually review from time of informed consent													
Concomitant Medications	Continually review from time of informed consent													
IMP Dispensing		X												
IMP Administration[2]		X	X	X	X	X	X	X	X	X	X			
WHO performance status (Appendix 2)	X													
WHO COVID Ordinal Severity Scale(Appendix 1)	X	X	X	X	X	X	X	X	X	X	X	X	X	X

Investigation	Screening (d-4 to day 1)	Day 1	Day 2	Day 3	Day 4	Day 5	Day 6	Day 7	Day 8	Day 9	Day 10	Day 15[5]	Follow-up(day 29)[6]	Follow-up (day 60)[6]
Routine Clinical Blood tests[3] (see table 2)	X	X		X					X			X	X	X
Blood Samples for Epigenetic and Immunological testing[3]		X[10]		X[10]								X	X	X
Viral PCR (nasopharyngeal swab)[3]	X[8]	X[4]		X					X [11]		X	X	X	X
Viral Load (nasopharyngeal swab)[3]		X[4]		X					X		X	X	X	X
Pregnancy Test[7]	X													
PK testing[9]		X		X		X			X		X			
COVID19 Health and Wellbeing Follow up Survey (Appendix 3)												X	X	X

1. Observations: Heart rate, temperature, oxygen saturations, blood pressure, respiratory rate
2. IMP should start to be administered within 24 hours of randomisation
3. Bloods/Nasopharyngeal swabs can be taken +/- 48 hours from scheduled time point unless otherwise specified. Screening bloods/NP swabs should be performed within 96 hours of randomisation
4. Nasopharyngeal swabs on day 1 should be taken as close to T0 (time of first drug administration) as possible
5. Patients discharged prior to day 15 should follow the outpatient schedule of assessments from point of discharge. Patients should return for clinic visit where possible, or be followed up by phone call if unable to attend

6. Follow-up visit at day 29 and 60 may be performed by telephone if patient is unable to attend clinic
7. Serum pregnancy test, for patients of child bearing potential only
8. ScreeningCOVID-19 test can be done using any valid method of testing
9. PK Samples should be taken at the following times:
    - Day 1: pre dose and 0.5 hours post dose
    - Day 3: pre and 0.5 hours post dose
    - Day 5: pre and0.5 hours post dose
    - Day 8: Pre dose
    - Day 10: Pre dose

    In addition, 16 volunteers (8 male, 8 female) will have more frequent sampling for PK on days 1 and 3 after either dose that day. Hours post dose: 0.5, 1, 2, 7, 12
    PK samples require to be spun within 4 hours of being taken – where sites do not have the facility to do this, PK's will be omitted.
10. Blood samples for epigenetic and immunological testing should be taken at the time of the first PK draws on days 1 and 3 (i.e. pre-dose)
11. Two NP swabs should be taken on day 8 – one for immediate COVID-19 testing (any valid method) and one to be stored for later viral load analysis.

Schedule of Events for outpatients

Investigation	Screening (d-4 to d1)	Day 1	Day 2	Day 3	Day 4	Day 5	Day 6	Day 7	Day 8	Day 9	Day 10	Day 15	Follow-up(day 29)[5]	Follow-up(day 60)[5]
Written informed consent	X													
Demographic and Medical History	X													
Randomisation	X													
Observations[1]	X											X	X	X
Height and weight	X											X	X	X
Physical Exam	X											Symptom directed where clinically indicated		
AE Evaluation	X	Continually review from time of consent [8]												
Concomitant Medications	X	Continually review from time of consent[8]												
IMP Dispensing[10]		X												
IMP Administration [2]		X	X	X	X	X	X	X	X	X	X			
WHO performance status (Appendix 2)	X													

Investigation	Screening (d-4 to d1)	Day 1	Day 2	Day 3	Day 4	Day 5	Day 6	Day 7	Day 8	Day 9	Day 10	Day 15	Follow-up(day 29)[5]	Follow-up(day 60)[5]
WHO COVID Ordinal Severity Scale (Appendix 1)[8]	X	X	X	X	X	X	X	X	X	X	X	X	X	X
Routine Clinical Blood tests[3] (see table 2)	X											X	X	X
Blood Sample for PK analysis[11] (favipiravir arm only)	X[11]	X[11]												
Blood Samples for Epigenetic and Immunological testing[3]	X											X	X	X
Viral PCR (nasopharyngeal swab)[3]	X[7]								X[9]			X	X	X
Viral Load (nasopharyngeal swab)[3]	X								X[9]			X	X	X
Pregnancy Test[6]	X													
COVID19 Health and Wellbeing Follow up Survey (Appendix 3)												X	X	X

1. Observations: Heart rate, temperature, oxygen saturations, blood pressure, respiratory rate
2. IMP should start to be administered within 24 hours of randomisation

3. Bloods/Nasopharyngeal swabs can be taken +/- 48 hours from scheduled time point unless otherwise specified. Screening bloods should be performed within 96 hours of randomisation
4. Nasopharyngeal swabs on day 1 should be taken as close to T0 (time of first drug administration) as possible
5. 5.Follow-up visit at day 15, 29 and may be performed by telephone if patient is unable to attend clinic
6. Serum pregnancy test, for patients of childbearing potential only. Result must be confirmed negative before IMP is released to patient.
7. Screening COVID-19 test can be done using any valid method of testing
8. AE, con med and WHO COVID Ordinal Severity Scale assessments should be made by phone call on days 1 – 10.
9. Optional nasopharyngeal swab may be done by patient at home if possible and safe to transport
10. To minimise the time spent in the clinic, patients can return home after screening and have IMP delivered to their home following randomisation
11. PK sampling should only be done where site have provision to take a predose sample (screening or day 1 pre-dose) AND a post dose sample (30 minutes after dosing), and have facilities to spin samples within 4 hours.

1 INTRODUCTION & BACKGROUND

Description of the proposed trial, patient population and disease area

The proposed trial will assess the efficacy of favipiravir as an early treatment for COVID-19. COVID-19 is an acute illness characterised principally by fever, cough and respiratory failure, caused by the Coronavirus SARS-CoV-2. It was reported for the first time in Wuhan in China at the end of 2019, and is now causing a pandemic. As of 12th April 2020 there were 78 995 confirmed cases in the UK, and 9875 deaths since the beginning of 2020. Globally the number of confirmed cases stood at 1, 696 588 with 105 952 deaths[3]

Current treatment for COVID-19 and IMP

Currently two medications have shown promise for treating severe COVID19: Dexamethasone {The, 2020, Dexamethasone in Hospitalized Patients with Covid-19 — Preliminary Report}and Remdesivir {Beigel, 2020, Remdesivir for the Treatment of Covid-19 - Preliminary Report. Reply}. No medication has yet been proved effective when started early when the disease is still milder. Several IMPs are currently in or about to start testing in clinical trials[6]. An efficient approach to finding effective treatments against COVID-19 is to test existing antiviral agents that have previously been shown to be effective against other viral infections, including other members of the coronaviridae family.

The GETAFIX trial is testing the use of favipiravir as treatment for COVID-19. Favipiravir (T-705) is a pyrazinecarboxamide derivative. It is a pro drug which is converted to nucleoside analogue within the cell of interest, followed a series of phosphorylations to produce the active ribofuranosyltriphosphate T-705RTP which selectively inhibits viral RNA-dependent RNA polymerase [7].

Non clinical studies with potential clinical significance

Favipiravir has shown potent *in vitro* inhibitory activity against seasonal influenza A (H1N1[2009], H2N2, H3N2, H5N1), avian influenza A (H7N9), influenza B, influenza C viruses, as well as against oseltamivir-resistant and amantadine-resistant strains[8].

It has also been shown to protect mice against Ebola virus Challenge (Oestereich *et al*. 2014) and has activity against RNA viruses including, Influenza, EBOV, arenaviruses [8-10] and filoviruses [2, 10]. *In vitro* studies have demonstrated activity against Ebola and other RNA viruses (for example, Lassa

fever [*Arenaviridae]*, *Bunyaviridae*, Chikungunya virus [*Alphavirus*]) via a similar mechanism of action [8].

Favipiravir effectively inhibits SARS-CoV-2 infection in Vero E6 cells (ATCC-1586) [7], The EC_{50} for COVID-19 *in vitro* is 61.88 µM, indicating an inhibitory effect (similar to that for Ebola virus) [11].

Previous use of Favipiravir in human patients

Favipiravir has been used in more than 40 clinical studies, mainly in the US and Japan (33 Phase I, 3 Phase II and 4 Phase III). It has a favourable safety profile with a low frequency of mild to moderate adverse events (AEs), notably mild to moderate, asymptomatic elevations in serum uric acid and mild to moderate diarrhoea. It has also been used to treat acute Ebola Virus Disease in Guinea in a clinical trial which was not significant but had promising trends (JIKI trial) [2], and has been used in a trial to treat Ebola Virus in Sierra Leone[1]. Previous use of favipiravir to treat patients with COVID-19 specifically is discussed below.

1.1 IMP: Prior Experience in COVID-19 and dose selection

There are limited, recent reports of the use of favipiravir for treatment of COVID-19 in the context of clinical trials performed in China. Cai *et al.* [5] reported the results of an open label Control study comparing favipiravir (35 patients) vs Lopinavir/Ritonavir (R) (45 patients) for the treatment of COVID-19. Both arms also received interferon-alpha by aerosol inhalation. Favipiravir shortened the viral clearance time from 11 days to 4, and showed significant improvement in chest imaging (91% vs. 62%). Patients taking favipiravir also had fewer adverse events than those taking Lopinavir/R. Chang Chen *et al*. 2020[12] have reported results from a prospective, multicentre, open-label randomised superiority trial comparing: favipiravir (N=116) to Arbidol (N=120). Clinical recovery within 7 days was 55.9% in the Arbidol group and 71.4% in the favipiravir group. This study concluded that favipiravir was the preferred treatment for non-severe COVID-19.

To date, six Chinese studies (other than the trial[13] that has already reported results by Cai *et al.*.[5]) exploring the use of favipiravir against COVID-19 have been posted to the Chinese Clinical Trial Registry website. [14-19]

A randomised (1:1) trial has been posted by researchers in Egypt to the clinicaltrials.gov website.[14] This trial will randomise patients to receive favipiravir (1600mg BD day 1 and 600mg BD days 2-10)

or standard of care therapy, which the investigators define as oseltamivir 75mg BD for 5-10 days and hydroxycholoroquine 400mg BD day 1 followed by 200mg once daily from day 2 to day 5-10. This trial has reported that it has completed recruitment but has not yet reported results.

Rationale for dose selection

In a clinical pharmacology study in which 1800mg of favipiravir was administered twice daily on Day 1 and 800mg of favipiravir was administered twice daily on Day 2 and thereafter (1800/800mg twice daily) for 22 days to healthy Japanese adult males aged 20 to 39 years, the peak plasma concentration of favipiravir remained at approximately 87 to 104µg/mL on Day 5 and after, and C_{min} remained at approximately 56 to 75µg/mL after the 2nd administration on Day 1. This value exceeds 9.72µg/mL, the EC_{50} of favipiravir for the SARS-CoV-2 virus and is significant even when the human protein binding rate of favipiravir of approximately 50% is taken into consideration. The Jiki trial[2] for Ebola, used a dose of 6000 mg on day 1, and 2400mg on days 2 to 10 and reported no grade 3 or 4 adverse events. The clinical trial that recruited from Sierra Leone used a dose of 1600mg twice daily on D1 and 600mg daily days 2-14[1].The EC50 for Ebola for favipiravir is slightly higher than for COVID-19 at 10.5µg/mL.

The licensed dose in Japan of favipiravir for severe influenza is 1600mg twice daily on day 1 followed by 600mg twice daily for 4 days and the Japanese Association for Infectious Diseases have recommended the dosage and administration of favipiravir for COVID-19 as 3600 mg (1800 mg twice daily Day 1) followed by 1600 mg (800 mg twice daily Day 2 and thereafter) administered for up to 14 days.

The dose of favipiravir used in the recently completed clinical trials for patients with COVID-19 was 1600mg twice daily on D1 and 600mg twice daily days 2-14 [5 12]. In the trial reported by Cai *et al.*, patients also received aerosolized interferon-alpha treatment (5 million units twice daily). The interquartile range of time for viral clearance was 2.5 to 9 days (median4) in the favipiravir plus aerosolised interferon arm compared to 8-13 days (median 11) in the control arm taking lopinavir/ritonavir.

Considering the previous available evidence, the dose proposed for COVID-19 in the GETAFIX trial is 3600mg on day one followed by 1800mg on days 2 to 10. This should maintain the EC_{50} above the

required concentration of 61.88microM (9.72microg/ml) and allow investigation of the efficacy of the single agent regimen.

1.2 Trial Rationale and Hypothesis

There is a current global pandemic of COVID-19 infection. This is caused by a novel virus which was first identified worldwide in December 2019 and has now been detected in every country globally. As of 19th July 2020 there were 294 070 confirmed cases in the UK, and 45 273 deaths since the beginning of 2020. Globally the number of confirmed cases stood at 14 043 611 with 597 583 deaths {World Health, 2020}.As well as this effect on health, it has had major healthcare service, economic and social repercussions. Currently two medications have shown promise for treating severe COVID19: Dexamethasone {The, 2020, Dexamethasone in Hospitalized Patients with Covid-19 — Preliminary Report}and Remdesivir {Beigel, 2020, Remdesivir for the Treatment of Covid-19 - Preliminary Report. Reply}. No medication has yet been proved effective when started early when the disease is still milder. Several IMPs are currently in or about to start testing in clinical trials[6].

Favipiravir is an antiviral agent that is already used against severe influenza (as outlined in the favipiravir Investigational Brochure) and that has been used in trials against other viruses with promising results. There is therefore a strong rationale for testing favipiravir against COVID-19 and there is an urgent need to do so in a controlled, clinical trial setting.

The working hypothesis for the GETAFIX trial is that favipiravir may be an effective treatment for COVID-19 infection for those patients who have early stage disease, compared to current standard of care. This hypothesis will be tested by evaluating the difference in severity of disease at day 15 as the trial primary endpoint. This study will also provide an important opportunity to evaluate the pharmacokinetic and pharmacodynamic profile of this drug in the context of COVID-19 infection. It is critical to gather this information alongside measures of clinical efficacy, to better understand the clinical outcomes and to provide information for ongoing trials and use of favipiravir. Experimental endpoints will include evaluation of potential mechanisms of resistance of COVID-19 to this drug. If successful at meeting its primary endpoint, the GETAFIX trial has the potential to be highly impactful if favipiravir is considered for use globally to treat COVID-19 to reduce deaths, admissions to hospital and transmission of SARS-CoV-2.

2 TRIAL OBJECTIVES

Table 1: Objectives and Endpoints

**Primary Objective**	**Primary Endpoint**	**Timepoint of assessment**
Assess the efficacy of favipiravir in addition to standard care in patients with COVID-19 in reducing the severity of disease compared to standard care alone	Clinical status as assessed by WHO COVID 10 point ordinal severity scale* at day 15	Day 15 (+/- 48 hours)
**Secondary objectives**	**Secondary Endpoints**	**Timepoint of assessment**
Assess the effect of favipiravir in addition to standard care in the study population compared to standard care alone	Proportion of patients meeting level 7 or above on the WHO COVID 10 point ordinal severity scale or dead by day 29<br><br>WHO COVID 10 point ordinal severity scale level<br><br>Overall survival<br><br>***Inpatients only:***<br>Viral clearance on or before d8 **<br><br>***Inpatients only:***<br>Duration of pyrexia in days (defined as a temperature ≥ 38ºC)	Up to and including day 29<br><br>Days 8, 29, 60<br><br>Up to and including day 60<br><br>Day 8<br><br>Up to and including day 60
Evaluate the safety and tolerability of favipiravir in the study population	Adverse events	Up to and including day 60
**Exploratory objectives**	**Exploratory endpoints**	**Timepoint of assessment**
Evaluate the effect of favipiravir on duration of hospitalisation	Duration of hospitalisation (days)	Up to and including day 60
Understand the pharmacokinetics of favipiravir	Evaluate the population pharmacokinetics of this dose of favipiravir in the study population and assess the relationship between the pharmacokinetics of favipiravir in the study population and viral load	Please see Appendix 5 for schedule of sampling

Understand the pharmacodynamics of favipiravir	Change in viral load with time<br><br>***Outpatients only***:<br>Viral clearance on or before d8 **	Up to and including day 60<br><br><br>Day 8
Understand mechanisms of resistance to favipiravir	Sequence of virus in cases of treatment failure	Up to and including day 60
Explore the immunological and biometric markers contributing to the clinical condition of patients in the study population	Evaluate the relationship between immunological and biometric markers and the clinical outcomes of the study	Up to and including day 60
Post COVID19 health and psycho-social consequences	Evaluate the relationship between post COVID-19 health and psycho-social consequences and the clinical outcomes of the study	Up to and including day 60

*See Appendix 1 for WHO COVID-19 10 point ordinal severity scale.

** Defined as a negative SARS-CoV-2 PCR result

3 TRIAL DESIGN

GETAFIX is a randomised controlled study of favipiravir as an early treatment arm in COVID-19 patients who are over 16 years old, not pregnant or breastfeeding and without previous co-morbidity. The study is a single-centre, randomized (1:1) open label phase II/III study which allows for early stopping in case of lack of efficacy or clear superiority in the experimental arm. Eligible patients will be randomised between standard care for COVID-19 (Control arm) and standard care plus favipiravir (Experimental arm).

The study is powered to detect an improvement corresponding to an odds ratio of 1.95 in the cumulative odds of the WHO COVID 10-point ordinal severity scale assessed at day 15. This is equivalent to the probability of a patient having a better outcome at this time if allocated to experimental as compared to control of 66% (50% under null hypothesis).

To detect this magnitude of difference with 90% power at the 5% 2-sided level of statistical significance requires 302 patients (151 per arm).

This trial will be performed according to the UK Policy Framework for Health and Social Care (2017) Medicines for Human Use (Clinical Trials) Regulations, 2004 SI 2004:1031 (as amended) and World

Medical Association Declaration of Helsinki Ethical Principles for Medical Research Involving Human Subjects 1964 (as amended)

3.1 Trial population

In total, 302 adult patients with confirmed COVID-19 by valid COVID-19 test will be recruited to the trial and randomised (1:1) across both the control arm and the experimental arm.

The study population for this trial will include patients aged ≥ 16 years old with a confirmed diagnosis of COVID-19, who are not pregnant or breastfeeding. Patients will be entered into this trial within 96 hours of the COVID-19 test being taken. Potentially eligible patients will be identified after community testing, in an acute receiving environment or when admitted to a hospital ward.

3.2 Eligibility criteria

Inclusion criteria

1. Age 16 or over at time of consent
2. Exhibiting symptoms associated with COVID-19
3. Positive for SARS-CoV-2 on valid COVID-19 test
4. Point 2, 3, or 4 on the WHO COVID-19 ordinal severity scale at time of randomisation. (Symptomatic Independent, Symptomatic assistance needed, Hospitalized, with no oxygen therapy)
5. Have >=10% risk of death should they be admitted to hospital as defined by the ISARIC4C risk index: https://isaric4c.net/risk
6. Able to provide written informed consent
7. Negative pregnancy test (women of childbearing potential*)
8. Able to swallow oral medication

Exclusion criteria

1. Renal impairment requiring dialysis or haemofiltration
2. Pregnant or breastfeeding
3. Of child bearing potential* (women), or with female partners of child bearing potential (men) who do not agree to use adequate contraceptive measures for the duration of the study and for 3 months after the completion of study treatment
4. History of hereditary xanthinuria

5. Xanthine urinary calculi
6. Other patients judged unsuitable by the Principal Investigator or sub-Investigator
7. Known hypersensitivity to favipiravir, its metabolites or any excipients
8. Severe co-morbidities including patients with severe hepatic impairment defined as:
    - greater than Child-Pugh grade A (Appendix 4)
    - AST or ALT > 5 x ULN
    - AST or ALT > 3x ULN and Total Bilirubin >2xULN
9. More than 96 hours since first positive COVID19 test was taken.
10. Unable to discontinue contra-indicated concomitant medications (section 6.7)

* Non-childbearing potential must be evidenced by fulfilling one of the following criteria at screening:

- Post-menopausal defined as aged more than 50 years and amenorrhoeic for at least 12 months following cessation of all exogenous hormonal treatments.
- Irreversible surgical sterilisation by hysterectomy, bilateral oophorectomy or bilateral salpingectomy but not tubal ligation

There will be no exception to the eligibility requirements at the time of registration/randomisation. Queries in relation to the eligibility criteria should be addressed via contact with the CTU prior to registration/randomisation. Patients are eligible for the trial if all the inclusion criteria are met and none of the exclusion criteria apply.

3.3 Identification of participants and consent

Potential participants may be identified before their COVID-19 test result is available and will be randomised within 96 hours of their COVID-19 test being taken. They may be identified either by clinical staff involved in their treatment as per standard of care, or by a clinical member of the GETAFIX trial team. COVID-19 testing centres may provide adverts to attendees who may contact the GETAFIX trial team if interested in participating. Following this, patients will be seen by a clinical member of the GETAFIX trial team who will explain to the patient that they may be potentially eligible for a clinical trial. If the patient is interested in hearing more about the trial, this option will be discussed further with the patient.

After receiving verbal and written information, patients will be given sufficient time to decide whether or not they would like to take part in the trial. All efforts will be made to ensure that patients understand the commitment required to fulfil the trial requirements and facilitate discussion with friends and relatives at the request of the patient. Patients will be told that their

participation is entirely voluntary and that they can leave the trial at any time without their standard care being affected. Patients will then be given the choice of whether they wish to participate. Signed participant consent must be obtained. For patients who have capacity to understand and give consent, but are unable to read the consent form or sign their name, witness consent may be given. The consent forms should also be signed by the person undertaking the consent procedure at site, who must be detailed on the delegation log as having this authorisation. The Principal Investigator is responsible for ensuring the designee is suitably qualified by training or experience to take informed consent, if consent is delegated to a designee.

After the patient has provided informed consent to participate in the trial, the CRUK CTU, Glasgow will provide the site with a screening number for the patient, and screening may commence. No screening activities related to the trial may be undertaken until informed consent has been obtained.

Once the patient has been deemed eligible for the trial by the PI or medically trained designee, 1:1 randomisation into the Control Arm or the Experimental Arm will occur. In order for randomisation to occur, the participating site must fax, telephone or email the CRUK Clinical Trials Unit, Glasgow.

There will be an optional consent form for patients who may want to contribute to more frequent PK and PD sampling. The maximum number of patients who will undergo this testing will be sixteen, eight males and eight females. This option will be offered to all patients who are being treated in hospital at the time of taking informed consent, however only patients in the experimental arm of the trial will be able to undergo this testing.

For those patients who are randomised to the experimental arm of the trial, it is strongly advised that the first dose of favipiravir is administered within 24 hours of the treating clinician receiving a positive SARS-CoV-2 test result.

3.4 Registration/Randomisation

Patients cannot be screened or registered to the trial until the site has been activated to begin recruitment. To register/randomise a patient on the trial, contact the CRUK Clinical Trials Unit, Glasgow. Registration/Randomisation to the trial can be performed by either telephone or fax on the following numbers:

Telephone Number: 0141 301 7246

Fax Number: 0141 301 7946

Email: getafix.crukglasgowctu@nhs.net

Registration/Randomisation by telephone or fax will only be available during the following hours:

Mon – Thurs 8.30am – 5pm

Fri 8.30am – 4.30pm

An out of hours service for registering/randomising inpatients will be operational at the following times:

Monday – Thursday from 5.00 pm- 6.00pm

Friday from 4.30pm -6.00pm

Saturday 9am – 4.00pm

Sunday 9am – 12.00pm

During these times, only randomisations sent by email will be processed.

Please check local site Pharmacy opening times – patients should be randomised no later than 1 hour before local site Pharmacy closes.

Out of hours randomisation availability is dependent on site Pharmacy and Nursing staff availability – this may differ between sites and at different times according to the progress of the pandemic. Sites will be kept informed of any changes to this service.

When the patient has consented, a screening number should first be obtained by contacting the CRUK CTU, Glasgow.

When screening is complete and eligibility confirmed, the CRUK CTU Glasgow should be contacted again. The patient's eligibility criteria will be checked and, if eligible, a trial number and trial arm will be allocated at this point.

All patients must be registered/randomised onto the trial prior to commencement of trial treatment.

A minimisation algorithm incorporating a random component will be used to allocate patients to either experimental or control arm. The factors used in the minimisation will be:

- Site
- Age (16 - 50; 51 – 70; 71+)
- History of hypertension or currently obese (BMI>30 or obesity clinically evident) (yes; no)
- <7 days duration of symptoms (yes; no; unknown)
- Sex (male; female).
- WHO COVID ordinal severity score at baseline (2 or 3; 4)

With the patient's consent, their GP will be informed of their involvement in the trial.

3.5 Withdrawal

3.5.1 Withdrawal of Patients from Trial Treatment

Patients have the right to withdraw from trial treatment at any point for any reason. Similarly, the investigator may withdraw patients from the trial drug in the event of intra-current illness, AEs, SAEs, SUSARs, protocol violations or any other relevant reasons**. If a patient withdraws from treatment early, they should be followed up as per trial schedule.**

A subject must be discontinued from the trial for any of the following reasons:

- The subject or legal representative withdraws consent
- Patient meets stopping rules criteria (see Section 6.8)
- Investigator's decision to withdraw the subject
- The subject has a confirmed positive serum pregnancy test
- Noncompliance with trial treatment or procedure requirements
- Administrative reasons

3.5.2 Withdrawal from Trial

In many circumstances where a patient withdraws from trial treatment, it will, nonetheless, be desirable for the patient to remain on the trial (for example for follow-up purposes). If, however, the patient withdraws from the trial itself, it should be clearly documented in the patient's notes what they are withdrawing from (consent to use any past data, consent to use any samples collected or consent for further data collection from the date of consent withdrawal). If a patient withdraws their consent from the trial, the site must contact the CRUK CTU Glasgow with full details of the withdrawal. Where applicable, the CRUK CTU Glasgow may ask the site to complete a Consent

Withdrawal Form to record full details of the consent withdrawal. In the event of withdrawal, data or tissue already collected with consent will be retained and used in the study, but no further tissue will be collected or any other research procedures carried out on or in relation to the participant.

Patients who become unwell to such an extent that they lose capacity during the course of the study will be permitted to continue on IMP until the point they are unable to swallow oral medication, and all other study processes, including safety assessments, should continue. No patient whether they have capacity or not will continue on the trial if it is unsafe to do so in the opinion of the Principal or Chief Investigator, or the Trial Steering Committee.

4 TRIAL PROCEDURES

4.1 Trial Endpoints

The trial endpoints are defined in Section 2.

4.2 Trial Schedule

Below is a list of expected assessments for each patient entered into the GETAFIX trial and the relevant time points. Assessments marked with asterisk (*) are considered part of standard care.

4.2.1 Schedule for Inpatients

Initiation of screening to first dose

Written informed consent

Assessment of eligibility criteria

Demographic details

Valid COVID-19 diagnostic test

Medical history including assessment of medical co-morbidities

Documentation of concomitant medications

Clinical examination (performed by Principal Investigator or their designee)

Human chorionic gonadotrophin (serum HCG) test to rule out pregnancy at trial entry; results must be obtained and reviewed before randomisation for women of childbearing potential.

Routine observations* (see Table of assessments for detail)

Routine clinical blood tests* (see Table 2)

Height and weight*

Pre-morbid performance status as assessed by WHO criteria (Appendix 2)

WHO COVID 10 point ordinal severity scale assessment (Appendix 1)

Day 1-10[1]

WHO COVID 10 point ordinal severity scale assessment (daily – worst value in last 24 hours)

Documentation of concomitant medications (daily)

Assessment of adverse events (daily)

Routine observations/vital signs* (daily)

Routine clinical blood tests: days 1,3,8,11 (+/- 48 hours)

Blood tests for immunological and epigenetic testing: days 1, 3 (+/- 48 hours)

Symptom directed clinical examination if clinically indicated

Administration of IMP (if applicable)

Nasopharyngeal swab (for SARS-CoV-2 PCR assessment and Viral Load): days 1 (at time of first dose), 3, 8[2], 10, 13 (+/- 48 hours)

Pharmacokinetic samples: according to the sample schedule (Appendix 5)

[1] Patients discharged during this time should follow the outpatient trial schedule. The timing of the first dose of favipiravir should be documented as Time zero (T0). The nasopharyngeal swab should be taken as close to this time as possible.

[2] On day 8, two swabs should be taken – one to be sent for immediate COVID-19 testing by any valid method, the other to be stored for later analysis of viral load.

Day 15[1]

Nasopharyngeal swab[2] (for SARS-CoV-2 PCR assessment and Viral Load) (+/- 48 hours)

WHO COVID 10 point ordinal severity scale assessment (Appendix 1).

Routine clinical blood tests[2]

Blood tests for immunological and epigenetic testing[2] (+/- 48 hours)

Pharmacokinetic samples- according to the sample schedule[2] (Appendix 5)

COVID-19 Health and Wellbeing Follow up Survey (Appendix 3)

[1] Day 15 assessment should be done in clinic if patient has been discharged. If patient is unable to attend clinic, a follow-up telephone assessment should be done.

[2] Optional if patient unable to attend clinic

Day 29 and Day 60[1]

Assessment of concomitant medication and adverse events to be undertaken by an investigator with the patient.[1]

Routine clinical blood tests[2]

Blood tests for immunological and epigenetic testing[2]

WHO COVID 10 point ordinal severity scale assessment (Appendix 1)

Nasopharyngeal swab[2] (for SARS-CoV-2 PCR assessment and Viral Load)

Concomitant medications

Assessment of adverse events

COVID-19 Health and Wellbeing Follow up Survey (Appendix 3)

[1] Day 29 and Day 60 assessments should be done in clinic if patient has been discharged. If patient is unable to attend clinic, a follow-up telephone assessment should be done.

[2] Optional if patient unable to attend clinic

4.2.2 Schedule for outpatients

Initiation of screening to first dose

Written informed consent

Assessment of eligibility criteria

Demographic details

Valid COVID-19 diagnostic test

Medical history including assessment of medical co-morbidities

Documentation of concomitant medications

Clinical examination (performed by Principal Investigator or their designee)

Human chorionic gonadotrophin (serum HCG) test to rule out pregnancy at trial entry; results must be obtained and reviewed before randomisation for women of childbearing potential.

Routine observations (see Table of assessments for detail)

Routine clinical blood tests (see Table 2)

Height and weight

Pre-morbid performance status as assessed by WHO criteria (Appendix 2)

WHO COVID 10 point ordinal severity scale assessment (Appendix 1)

Baseline PK sample[4]

Day 1-10[1]

Dispensing of IMP[2] (patients on favipiravir arm)

WHO COVID 10 point ordinal severity scale assessment (daily – worst value in last 24 hours)

Documentation of concomitant medications (daily)

Assessment of adverse events (daily)

Nasopharyngeal swab[3] (for SARS-CoV-2 PCR assessment and viral load): day 8

Administration of IMP and completion of Patient Diary (patients on favipiravir arm)

1 Assessments on day 1-10 will be performed by telephone

2 IMP may be dispensed in clinic following randomisation where possible, or couriered to the patient's home

3 NP swab may be performed at home by the patient provided this is safe to do and safe transport to research laboratory has been arranged.

4 Baseline PK sample can be taken at screening or pre-dose day 1. PK sampling will only be undertaken where sites can accommodate both pre and post dose sampling, and have the facilities to spin samples within 4 hours.

Day 15[1]

Nasopharyngeal swab[2] (for SARS-CoV-2 PCR assessment and Viral Load) (+/- 48 hours)

WHO COVID 10 point ordinal severity scale assessment (Appendix 1).

Routine clinical blood tests[2]

Blood tests for immunological and epigenetic testing[2] (+/- 48 hours)

End of Treatment Compliance check[3] - return of any remaining IMP/empty container, and completed diary (favipiravir arm only)

COVID-19 Health and Wellbeing Follow up Survey (Appendix 3)

[1] Day 15 assessment should be done in clinic - if patient is unable to attend clinic, a follow-up telephone assessment should be done.

[2] Optional if patient unable to attend clinic

[3] Compliance check should be done at next follow-up visit if patient cannot attend on day 15

Day 29 and Day 60[1]

Assessment of concomitant medication and adverse events to be undertaken by an investigator with the patient.[2]

Routine clinical blood tests[2]

Blood tests for immunological and epigenetic testing[2]

WHO COVID 10 point ordinal severity scale assessment (Appendix 1)

Nasopharyngeal swab[2] (for SARS-CoV-2 PCR assessment and Viral Load)

Concomitant medications

Assessment of adverse events

COVID-19 Health and Wellbeing Follow up Survey (Appendix 3)

[1] Day 29 and Day 60 assessments should be done in clinic if patient has been discharged. If patient is unable to attend clinic, a follow-up telephone assessment should be done.

[2] Optional if patient unable to attend clinic

Table 2: Routine clinical blood tests

Haematology	Biochemistry
Haemoglobin	Urea
WCC and differential	Creatinine
Haematocrit	Potassium
Platelets	Sodium
	AST
APTT	ALT
PT	Alkaline phosphatase
PT ratio	Bilirubin
APTT ratio	Albumin
Thrombin time	Uric acid
TCT ratio	Magnesium

Fibrinogen<br>D-Dimer<br>Ferritin	Calcium<br>Phosphate<br>Chloride<br>Bicarbonate<br>CRP<br>NT proBNP<br>High Sensitivity Troponin<br>HbA1c<br>Lipids<br>HDL-c<br>Anti-DNA/ANA

4.3 Laboratory Tests

4.3.1 Routine blood tests

Routine blood tests will be collected at site and sent to local NHS laboratories for processing. All laboratory tests will be conducted according to local procedures.

4.3.2 Valid COVID19 Test for diagnosis and viral clearance

An appropriate sample (usually a nasopharyngeal swab) will be collected and sent to local NHS laboratories for processing for diagnosis. Viral clearance on day 8 for inpatients will also be processed at a local NHS laboratory. Viral clearance at day 8 for outpatients will be collected at home by patients and sent to local academic laboratories for processing, provided it is feasible and safe to do so.

4.3.3 Viral Load for pharmacodynamics

A nasopharyngeal swab swill be collected and sent to local Academic laboratories for processing. Viral PCR testing will be conducted according to local procedures.

4.3.4 Pharmacokinetic/Pharmacodynamic sampling

Immunological, transcriptomic and epigenetic markers and plasma concentration of favipiravir for pharmacokinetic analysis : 1 tubes of EDTA blood and a Paxgene tube will be sent to local academic and NHS laboratories for safe processing and aliquots processed to generate plasma, PBMC and a whole blood layer which will be stored for experimental endpoints: immunological, epigenetic and

PK). The Paxgene tube will be stored for subsequent RNA extraction, RNA sequencing and BCR/TCR sequencing.
The schedule for PK/PD sampling is detailed in Appendix 5. Patients will only undergo PK sampling where the site has adequate facilities for processing of samples. Patients attending outpatient clinics will only undergo PK sampling where the site has the facility to accommodate both a baseline sample (screening or day 1 pre-dose) and a post dose sample following dosing on day 1.
For details of sample collection, processing and transfer for all of the above, please refer to Laboratory Manual.

5 TREATMENTS

For the purposes of this trial, favipiravir is considered an Investigational Medicinal Product (IMP).

5.1 Treatment Arms

Patients who are eligible for this trial will be registered/randomised to receive either:

<u>Control Arm:</u> Standard Treatment for COVID-19 infection

OR

<u>Experimental Arm:</u> Standard Treatment for COVID-19 plus favipiravir:

- Day 1: Loading Dose favipiravir 1800mg (9 x 200mg tablets) 12 hours apart (2 doses)
- Days 2 -10: Maintenance Dose favipiravir 800mg (4 x 200mg tablets) every 12hours (18 doses).

Subjects entering the study with a Child-Pugh liver impairment of grade A should receive the same loading dose but a reduced maintenance dose of favipiravir 600 mg twice daily on Days 2 through 10.

It is strongly advised that the first dose of favipiravir is administered within 24 hours of the treating clinician receiving a positive valid SARS-CoV-2 test result.

Treatment to continue until complete, unless there are unacceptable toxicities or other appropriate reasons for stopping treatment before completion.

5.2 Specific Drug Information

5.2.1 Favipiravir

Favipiravir is considered IMP for the purposes of this trial.

Chemical structure of favipiravir:

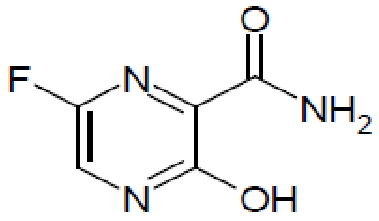

Favipiravir is supplied as light yellow round film-coated tablets at the dosage strength of 200mg per tablet. They are supplied in 100 tablet packs containing tablets in blister packaging. Refer to favipiravir Investigator Brochure for information regarding the physical and chemical properties and a list of excipients. Favipiravir should be stored at 15° to 30°C and shielded from direct light and moisture.

5.3 Dispensing, Accountability and Administration

The investigator or a delegated individual (e.g. pharmacist) must ensure that the trial IMP are dispensed in accordance with the protocol, local Standard Operating Procedures and applicable regulatory requirements.

5.3.1 Favipiravir Dispensing and Accountability

Batch number, dose prescribed, quantity and expiry of the drug supplied must be recorded in the accountability logs. Both bulk stock and individual patient accountability logs must be completed. Favipiravir course should be dispensed for inpatient use in a container and with instructions applied that make it suitable for use at the time of discharge, should continuation be required after discharged to complete the course. Full instructions regarding management, labelling and accountability of favipiravir is given in the IMP Management Document for the trial.

5.3.2 Favipiravir Administration

Favipiravir should be administered every 12 hours with no consideration of timing required in relation to food. If the patient vomits at any time after administration, dosage should not be replaced. If a dose is missed, it can be taken within 4 hours of the planned time and the next dose should be taken at the original dosing planned dosing time. After this time the missed dose should not be replaced. Favipiravir is not suitable for nasogastric administration.

Favipiravir may cause some people to become more sensitive to light and cause a rash or other mild skin reaction. Patients should be advised to avoid strong sunlight or UV light (e.g. a sun bed) during and for 1 week after favipiravir treatment, and to cover any sun exposed areas of skin and use sunscreen with high sun protection factor (SPF).

It is strongly advised that the first dose of favipiravir is administered within 24 hours of the treating clinician receiving a positive SARS-CoV-2 test result.

If a patient on the Experimental Arm of the trial is no longer able to take favipiravir because they are no longer able to swallow tablets (for example, if they are being treated with mechanical ventilation), all trial assessments should continue whenever possible.

5.4 Trial Drug Supplies

5.4.1 Favipiravir Supplies

Favipiravir will be provided free of charge from FUJIFILM Toyama Chemical Co., Ltd. to sites via Clinigen Group Plc for use by patients in this trial and will be commercial stock from Japan over-labelled as trial specific investigational medicinal product trial stock.

- Favipiravir will be packaged into boxes containing 100 tablets packs containing tablets in blister packaging. The supplies will be in commercial livery as licensed stock from Japan, with the details in Japanese. Clinigen will haveapplied labels that comply with Good Manufacturing Practice and in accordance with local regulatory labelling requirements (Annex 13 of Eudralex Volume 4). The labels will state that the trial dug is for clinical trial use only
- Only those supplies intended for use in the trial should be dispensed to the trial participants and clinical trial supplies must be dispensed in accordance with the trial protocol

- Upon arrival of investigational product at site, site personnel should check the supplies for damage and verify proper identity, quantity, integrity of seals and report any deviations or product complaints to the CRUK CTU upon discovery
- All trial medications must be stored in a secure area with access limited to the investigator and authorised site staff and under physical conditions that are consistent with the specific requirements of the medication. Favipiravir should be stored at 15° to 30°C and shielded from direct light. For batch specific instructions and information on the shelf life of favipiravir see the packaging.
- Full instructions regarding management, labelling and accountability of favipiravir is given in a separate IMP Management Document for the trial

5.5 Trial Treatment Recording

Reasons for any dose delays, dose reductions and dose omissions of trial drugs should be documented in the appropriate section of the CRF and in the patient's medical records

For patients self-administering favipiravir at home, a diary will be provided to record the time each dose was taken, and the reason for any missed doses – patients will be asked to return the completed diary along with any remaining drug or empty containers at a follow-up clinic visit.

5.6 Concomitant Therapy

All concomitant therapy taken at any point from 4 weeks before registration/randomisation up to and including the end of treatment visit must be documented in the patient's medical records along with dose, frequency and therapeutic indication.

5.7 Prohibited Therapy/Prohibited Medications/Permitted Concomitant Medications

- Other medication taken for treatment of COVID-19 whether investigational or approved may be permitted on a case by case basis – please contact Sponsor & CI to discuss.
- When remdesivir is added to the UK protocols as part of the standard of care for treatment of COVID19, co-prescribing will be permitted with favipiravir with regular monitoring of LFTs according to the combined protocol schedules for both medications. remdesivir prescription must be recorded in the patient CRF.
- Dexamethasone has been shown to have benefit in the treatment of COVID-19 in certain groups of patients and is being adopted into standard care. Treatment with

dexamethasone is permitted concurrently with favipiravir. Coadministration of dexamethasone with favipiravir has not been studied. Dexamethasone is a substrate of CYP3A4 and a moderate inducer of this enzyme. Favipiravir does not appear to be metabolised by this pathway – but there is limited data and Investigators must be vigilant to the possibility of interactions. Dexamethasone prescription must be recorded in the patient CRF.

Details of the administration of any prohibited therapies will be collected on the trial CRFs and must be reported as a protocol deviation as per section 11.3.

5.7.1 Interactions with other medications

There have been limited direct interaction studies of favipiravir and there is limited clinical experience of wide use of the product. Investigators must be vigilant to the possibility of interactions with other medicines other than those listed below.

- Paracetamol AUC may be increased and dosage should be restricted to 3g in 24h – this may require to be lowered further for lower bodyweight patients. This should be highlighted on the patient's notes, and clear instruction given to patients treated in the community.

The following may interact with favipiravir and risks and benefits should be carefully considered prior to treatment with the following:

- Any other anti-viral medication whether investigational or approved.
- Any drugs known to significantly inhibit Aldehyde Oxidase activity (e.g., pyrazinamide, amitriptyline, chlorpromazine, clomipramine, clozapine, erythromycin, ketoconazole, nortriptyline, quetiapine, raloxifene, perphenazine, promethazine, propafenone, tamoxifen, thioridazine).

Any drugs metabolized by the Aldehyde Oxidase pathway (e.g., famciclovir, hydralazine, lamivudine, sulindac, zaleplon, ziprasidone).

- Any drugs that interact with xanthine oxidase pathway, e.g. theophylline and aminophylline.
- Any drugs metabolised by CYP2C8, e.g. concentration of repaglinide concentration may be increased.

- Drugs with possible drug-drug interactions (concomitant medications requiring particular attention – see the Investigator Brochure).

This list is not exhaustive and IB should be checked in addition.

5.7.2 Adverse Reactions

Adverse reactions have been noted in the US and Japanese clinical studies where lower doses and shorter durations of treatment were used than in this protocol. The most common reactions included increases in blood uric acid levels, nausea, vomiting, diarrhoea, headache, ALT&AST increases, UTI, decreases in neutrophil and white blood cell count and increases in blood triglycerides.

Further details can be found in the Investigator's Brochure.

5.8 Dose Modifications for toxicity

A subject should be removed from favipiravir treatment if one of the following criteria is met:

- AST or ALT > 5 x ULN
- ALT or AST > 3 x ULN AND total bilirubin > 2X ULN
- AST or ALT > 3 x ULN AND patient has right upper quadrant pain or eosinophilia
- Uric acid >20 mg/dL (1.2 mmol/l)
- Any other ≥ Grade 3 adverse event considered by the study team to be due to the trial drug.

Any > 3 x ULN AST or ALT event should be reported as a AE of Special Interest using the SAE form provided by the CRUK CTU Pharmacovigilance department.

No dose reductions are required for reductions in renal function if they occur during treatment. There no data for treatment with favipiravir during haemofiltration or dialysis – if this occurs during treatment the Sponsor & CI should be contacted to discuss the risk-benefit of continuing treatment for the individual case.

If remdesivir is administered in combination with favipiravir, EGFR must be >30ml/min and LFTs must be monitored daily during treatment with remdesivir. The stopping rules above for raised

transaminases apply for the combination treatment. Ensure remdesivir SmPC or equivalent source of information is consulted before initiating the combination treatment with favipiravir.

5.8.1 Treatment Interruptions

No planned treatment interruptions are permitted.

5.8.2 Dose Modifications during Favipiravir Treatment

No dose modifications once on treatment are permitted for toxicity. Please see instructions for stopping in section 6.8.

5.9 Participation in concurrent clinical trials

Patients participating simultaneously in another clinical trial of investigational medicinal product (CTIMP) at the time of enrolment to GETAFIX may participate if the IMP in the other trial is not directly contraindicated in combination with favipiravir; it is not expected to interact with favipiravir based on available information and the Sponsor of the other CTIMP gives explicit permission and instruction for the withholding of the other IMP during participation in GETAFIX, if appropriate.

Patients will be permitted to take part in observational research at any time whilst participating in this trial.

Patients who have attained the primary trial end-point and are no longer receiving the trial intervention are permitted to enrol in other trials for COVID-19 subsequent to their participation in GETAFIX, subject to the inclusion criteria of those trials.

If a consideration is required that is not detailed above, the Principal Investigator seeking the consideration should seek approval from the Chief Investigator (CI) and Sponsor prior to enrolling the patient in either GETAFIX or the other trial. It is imperative that the Sponsor(s) of the both studies approve co-enrolment within their respective trials.

5.10 Duration of Trial Participation

5.10.1 Duration of Trial Treatment

Treatment with favipiravir is for 10 days only unless the participant experiences unacceptable toxicity or the treating clinician and patient decide it is in the patient's best interest to stop treatment sooner.

5.10.2 Duration of Trial Follow-up

All randomised participants will be followed up until day 60.

6 SAFETY REPORTING

Safety reporting will be performed by the Pharmacovigilance Department of the CRUK CTU Glasgow as delegated by the trial Sponsor

6.1 Pharmacovigilance

6.1.1 Definitions

Term	Definition
**Adverse Event (AE)**	An adverse event (AE) is any untoward medical occurrence in a subject to whom a medicinal product is administered, which does not necessarily have a causal relationship with this treatment.
**Adverse Reaction (AR)**	An adverse reaction (AR) is any untoward and unintended occurrence in a subject to whom a medicinal product has been administered which is thought to be caused by or related to that product.
**Serious Adverse Event (SAE)**	A serious adverse event (SAE) means any untoward medical occurrence that at any dose requires the following, whether or not considered related to the trial treatment.<br>• Requires inpatient hospitalisation or prolongation of existing hospitalisation*<br>• Results in persistent or significant disability or incapacity<br>• Results in a congenital anomaly/birth defect<br>• Is life threatening (i.e. at the time of the event)**<br>• Or results in death<br>• Is considered medically significant by the Investigator***<br><br>*Requires in-patient hospitalisation should be defined as a hospital admission required for treatment of an AE. No time frame is specified for the duration of the admission.<br>**Life threatening means that the patient was at <u>immediate risk of death</u> from the event as it occurred. It does not include an event that, had it occurred in a more serious form, might have caused death.<br>***Considered medically significant by the Investigator are events that may not result in death, are not life threatening, or do not require hospitalisation, but may be considered a serious adverse experience when, based upon appropriate medical judgement, the event may jeopardise the patient and may require medical or surgical intervention to prevent one of the outcomes listed above.

<table>
<tr><th>Term</th><th>Definition</th></tr>
<tr><td></td><td>Medical and scientific judgement should be exercised in deciding whether an event is “serious” in accordance with this criterion</td></tr>
<tr><td>Serious Adverse Reaction (SAR)</td><td>A serious adverse reaction (SAR) is a SAE that may be related to trial treatment. The assessment of “relatedness” is primarily the responsibility of the Principal Investigator (PI) at site or agreed designee. SAEs will be considered related if the SAE is documented as possibly, probably or definitely related to protocol treatment. The assessment of relatedness is made using the following:
<table>
<tr><th>Relationship</th><th>Description</th></tr>
<tr><td>Unrelated</td><td>There is no evidence of any causal relationship.</td></tr>
<tr><td>Possible</td><td>There is some evidence to suggest a causal relationship (e, g. the event occurs within a reasonable time after administration of the trial medication). However, the influence of other factors may have contributed to the event (e.g. the patient’s clinical condition, other concomitant treatments).</td></tr>
<tr><td>Probable</td><td>There is evidence to suggest a causal relationship and the influence of other factors in unlikely.</td></tr>
<tr><td>Definitely</td><td>There is clear evidence to suggest a causal relationship and other possible contributing factors can be ruled out.</td></tr>
</table>
</td></tr>
<tr><td>Unexpected Serious Adverse Reaction (known as a SUSAR</td><td>An Unexpected Serious Adverse Reaction means a serious adverse reaction, the nature and severity or outcome of which is not consistent with the reference safety information (RSI).</td></tr>
</table>

As some study related screening procedures are non-routine and potentially invasive, the risk of Adverse Events (AEs) and Serious Adverse Events (SAEs) occurring after consent to participate in the trial and before starting trial treatment, has been assessed as high. Therefore, these definitions apply to all trial participants from consent until day 60.

N.B: To avoid confusion or misunderstanding of the difference between the terms “serious” and “severe”, the following note of clarification is provided: “Severe” is often used to describe intensity of a specific event, which may be of relatively minor medical significance. “Seriousness” is the regulatory definition supplied above.

6.1.2 Detecting, Recording and Reporting of Adverse Events

6.1.2.1 Detection of Adverse Events

Participants will be asked at each trial visit about the occurrence of AEs since their last visit.

AEs will be recorded, notified, assessed, reported, analysed and managed in accordance with the Medicines for Human Use (Clinical Trials) Regulations 2004 (as amended) and the trial protocol.

AEs must be recorded as they are reported, whether spontaneously volunteered or in response to questioning about well-being at trial visits. The questioning about AEs will cover the current visit as well as the period between the previous and the current visit. All AEs must be documented in full in the patient's medical records whether they are required to be recorded in the CRF or not.

6.1.2.2 Recording of Adverse Events

Full details of AEs including the nature of the event, including the event term, start and stop dates, severity, seriousness and causality (relationship of the AE to each trial IMP) and outcome will be recorded in the patient's medical records. All AEs must be reported from the start of treatment and followed until:

- They resolve
- If present at pre-treatment, until the AE returns to the level observed at that time
- The AE is confirmed as unlikely to ever resolve

If none of the criteria above are met by follow-up day 29,the AE no longer requires to be followed up. Perceived lack of efficacy is not an AE.

An exacerbation of a pre-existing condition is an AE.

The Investigator does not need to actively monitor patients for AEs once the trial has ended, unless required.

Adverse events occurring in the period between consent and randomisation do not require to be recorded in the CRF if they are unrelated to any study specific screening procedure.

6.1.3 Assessment of Adverse Events

AEs must be assessed for seriousness, causality and severity. This assessment is the responsibility of the PI (or designee).

In determining whether an AE is an adverse reaction, Investigators must consider if there is a reasonable possibility of establishing a causal relationship between the event and favipiravir based on their analysis of all the available evidence. The assessment must be made on the basis of

anticipated pharmacological properties of favipiravir as specified in the IB or related to the patient's disease, either the disease under investigations or a concurrent illness.

The investigator must, whenever possible, provide a causality assessment for AEs based on the information available at reporting and their knowledge of the disease and favipiravir. The causality assessment provided by an Investigator shall not be downgraded by the CI.

**Relationship**	**Description**
**Unrelated**	There is no evidence of any causal relationship.
**Possible**	There is some evidence to suggest a causal relationship (e, g. the event occurs within a reasonable time after administration of the trial medication). However, the influence of other factors may have contributed to the event (e.g. the patient's clinical condition, other concomitant treatments).
**Probable**	There is evidence to suggest a causal relationship and the influence of other factors in unlikely.
**Definitely**	There is clear evidence to suggest a causal relationship and other possible contributing factors can be ruled out.

6.1.4 Reporting of a Serious Adverse Event

Investigators must report all Serious Adverse Events (SAEs) to the Pharmacovigilance Office, CRUK CTU immediately and under no circumstances should this exceed 24 hours following first awareness of the event by the Investigator or site staff.

In addition, all incidences of AT (ALT or AST) elevation > 3 times upper limit of normal (ULN) are classed as adverse events of special interest and should be reported as SAEs within the timeframe stated above.

The following events are excluded from reporting as SAEs

- Elective hospitalisations
- Day case admissions for treatment of underlying disease
- A and E attendance for an event that is not immediately life threatening and does not result in admission

Serious Adverse Events occurring in the period between consent and randomisation do not require to be reported if they are unrelated to any study specific screening procedure.

SAEs can be reported via the following means:
Pharmacovigilance Fax: 0141 232 2157
Email: mvls-ctu-pv@glasgow.ac.uk

For any guidance on SAE reporting please contact the Pharmacovigilance team on the following numbers:
Telephone: 0141 211 3567/0203/3969 or 232 2068

The purpose of this obligation is to ensure the CI on behalf of the Sponsor, has the necessary information to continuously assess the benefit-risk balance of the clinical trial.

For guidance on submitting and completing the initial and follow up SAE report forms please refer to the SAE report form Completion Guidelines, which will be provided by the Pharmacovigilance Office, CRUK CTU, Glasgow.
The CI will receive notification, by email, of all SAEs received.
SAEs must be reported locally by the PI at each site in accordance with the local practice at their site (i.e. R&D Office).

A follow-up report must be submitted when the SAE resolves, is unlikely to change, or as soon as additional information becomes available. If the SAE is a potential SUSAR then follow up information must be provided as quickly as possible and in the timeframe requested by the CRUK CTU and CI. All follow-up information is required to be reported promptly and follow up reports submitted until all AEs listed on the initial SAE report resolve. A follow-up report must be submitted if additional AEs listed on the initial SAE report have resolved or will never resolve. A follow-up report should also be submitted if additional AEs occur or new information becomes available about previously reported AEs.

All SAEs are required to be reported from start of treatment and for up to follow-up day 60. Any event that meets the criteria of a SAE (including events that the Investigator thinks are medically significant but maybe do not require hospitalisation or are fatal etc.) that occur after day 60 follow-up are also required to be reported if the Investigator thinks that the SAE is related to protocol treatment. Such SAEs must be reported to the Pharmacovigilance office without undue delay.

For any questions relating to SAE reporting, please contact the Pharmacovigilance team:
Email: mvls-ctu-pv@glasgow.ac.uk
Telephone: 0141 211 3569/0203/3969 or 232 2068
Contact details are also provided at the front of the protocol and in the SAE report form completion guidelines.

6.1.5 Identifying SUSARs

The assessment of expectedness for SAEs and regulatory reporting will be undertaken by the CRUK CTU Glasgow Pharmacovigilance office and CI. Expectedness assessments are based on the section of the trial Reference Safety Information (RSI) that has regulatory approval within the DSUR reporting period (and keeping the RSI the same throughout each reporting period) at the time the SAE is received.

When deciding if an event is unexpected consideration will be made by the CI as to whether the event adds significant information of the specificity, increase of occurrence or severity of a known, serious adverse reaction that is already recognised and documented in the RSI. Please note that for unlicensed IMPs any SAR that is fatal or life-threatening will automatically be reported as a SUSAR whether recorded in the RSI as an AE with fatal outcome, or not.

6.1.6 Reporting of a SUSAR

CRUK CTU on behalf of the (Co) Sponsor is responsible for the expedited reporting of all SUSARs to the required Regulatory Authorities, Research Ethics Committee, PIs at trial sites and the trial Sponsor(s) as well as pharmaceutical company (if applicable). The reporting of SUSARs starts with the authorisation of the clinical trial by any Regulatory Authority in the EU. It ends with the completion of the trial for all patients recruited (from the EU).

- Fatal or life threatening SUSARs will be reported within 7 days of the CRUK CTU receiving the first notification of the unexpected event. Any additional information will be reported within eight days of sending the initial report
- All other SUSARs will be reported within 15 days of the CRUK CTU receiving the first notification of the unexpected reaction
- SUSARs which initially are considered to be non-fatal or non-life threatening but which turn out to be fatal or life-threatening, will be reported as soon as possible and no later than 7 days after the CRUK CTU becoming aware of the reaction being fatal or life-threatening. If this report is incomplete a completed follow-up report will be submitted within an additional 8 days.
- SUSARs which initially are considered to be non-fatal or non-life threatening but which turn out to be fatal or life-threatening, if the initial report has yet to be submitted, will have a combined report submitted

If the assessment of causality provided by the investigator differs from that of the CI (assessment is made on behalf of the sponsor), the opinion of both the investigator and CI will be provided in the SUSAR report.

Investigators will be informed of all SUSARs as they are reported.

The CI will assess if the risk-benefit assessment has been affected by each SUSAR they identify. If the risk-benefit of participation is adversely affected, appropriate prompt action will be decided upon by the CI, Sponsor and Trial Steering Group and implemented by the Trial Management Group.

6.1.7 Other Unexpected Events Requiring Regulatory Reporting

In addition to SUSARs there may be other events that are relevant in terms of the risk-benefit balance. In addition to SAEs, SARs and SUSARs, all unexpected events that might materially influence the risk-benefit assessment of the IMP or that would lead to changes in the administration of favipiravir or in the overall conduct of a trial will also be reported to the Regulatory Authorities. This includes events which:

- Could be associated with trial procedures which could modify the conduct of the trial
- May be an increase in the rate of occurrence of an expected SAR which may be clinically important

- Potentially could be a significant hazard to the patient population such as a lack of efficacy of the IMP
- Are major safety findings from a newly completed studies (including animal studies)
- Would include the temporary halt of a trial for safety reasons in a trial with the same IMP also conducted by the Sponsor

Other unexpected events must be reported to the required Regulatory Authorities without delay, by the appropriate method (as an urgent safety measure, substantial amendment or early termination of the trial). Regulatory Authorities, Ethics Committees and the Sponsor will be informed of any safety issues that might materially alter the current benefit-risk assessment of the IMP while not falling into the actions for SUSARs noted above.

6.2 Pregnancy

Pregnancy occurring in a clinical trial participant, or the partner of a participant, whilst not considered an AE or a SAE, requires monitoring and follow-up.
Investigators must collect pregnancy information for female trial subjects or female partners of trial subjects. This includes subjects who become pregnant while participating in a clinical trial of an IMP or during a stage where the foetus could have been exposed to the IMP.

6.2.1.1 Contraception Requirements

- Male patients should refrain from fathering a child or donating sperm during the trial and for 3 months after the last administration of favipiravir.
- Patients of childbearing potential and their partners, who are sexually active, must agree to the use of 2 highly effective forms of contraception throughout their participation in the trial and for 3 months after the last administration of favipiravir.

This should be done as part of the consent process by explaining clearly to the patient the potential dangers of becoming pregnant and also providing each patient with information about appropriate medically approved contraception. Two forms of medically approved contraception should be used, such as:

Condom with spermicide, and 1 of the following:

- Oral contraceptive or hormonal therapy (e.g. hormone implants)

- Placement of an intra-uterine device

Acceptable hormonal methods:

- Etonogestrel implants (e.g., Implanon, Norplan) + male condom with spermicide
- Normal and low dose combined oral pills + male condom with spermicide
- Norelgestromin/ethinyl estradiol (EE) transdermal system + male condom with spermicide
- Intravaginal device + male condom with spermicide (e.g., EE and etonogestrel)
- Cerazette (desogestrel) + male condom with spermicide. Cerazette is currently the only highly efficacious progesterone based pill.

In certain restricted circumstances non-hormonal birth control methods are acceptable. These are detailed below:

Acceptable non-hormonal birth control methods include:

- Total sexual abstinence. This must be true abstinence which is in line with the usual preferred lifestyle of the participant (i.e. the participant should not become abstinent just for the purpose of inclusion into the clinical trial).
- Vasectomised sexual partner plus male condom. With participant assurance that partner received post-vasectomy confirmation of azoospermia.
- Tubal occlusion plus male condom with spermicide
- Intrauterine Device plus male condom + spermicide. Provided coils are copper-banded

Contraception should be effective before Day 1 of IMP administration, throughout the trial and for 3 months after completing the trial. It should be explained to male patients that if their partner is pregnant or breast-feeding when the patients enters the trial, the patient should use barrier method of contraception to prevent the unborn baby or the baby being exposed to favipiravir.

6.2.1.2 Pregnancy Reporting Requirements

The Investigator must ensure that all patients are aware at the start of a clinical trial of the importance of reporting all pregnancies (in themselves and their partners) that occur whilst being treated with favipiravir and occurring up to 3 months after the last IMP administration. The Investigator should offer counselling to the patient and/or the partner, and discuss the risks of continuing with the pregnancy and the possible side effects on the foetus. Monitoring of the

patient and the baby should continue until the conclusion of the pregnancy, if the patient or the patient's partner has consented to this.

If a patient or a partner of a patient does become pregnant, the reporting procedures below must be followed:

Any pregnancy occurring in a patient or a patient's partner during treatment with favipiravir) or occurring within 3 months of last administration of favipiravir must be reported to the Pharmacovigilance Office within 24 hours of the site staff becoming aware of it by completing the pregnancy eCRF and following this up, by, immediately faxing or emailing a completed Pregnancy Notification Form (PNF).

It is the Investigator's responsibility to obtain consent from the patient or patient's partner for following-up the pregnancy until outcome. The Pharmacovigilance Department of the CTU will follow-up all pregnancies until the pregnancy outcome via the Investigator, using the PNF.

The Investigator must update the PNF with the outcome of the delivery or if there is a change in the subject's condition such as miscarriage or planned termination. The updated PNF must be faxed or emailed to the PV office as soon as the information becomes available and no later than within 24 hours of first becoming aware of the change in condition.

Any pregnancies which result in a congenital anomaly or birth defect will require to be reported by the Investigator as a SAE. The Pharmacovigilance department will assist with providing guidance on reporting pregnancy outcomes as SAEs. SAEs that are the result of a birth defect will be reported as SUSARs if the CI and Sponsor consider the birth defect to be unexpected.

6.2.2 Medication Errors and Overdose

Medication errors, while not considered an AE or a SAE, may require monitoring and follow-up. Medication errors that result in SAEs should be reported as SAEs.

An overdose is defined as any dose greater than the highest daily dose included in this document. Any overdose must be recorded. If the overdose is associated with an AE, that AE must be recorded, assessed for seriousness, and reported as an SAE.

Any medication overdoses should be reported to the CRUK CTU Project Manager and Sponsor Pharmacist.

6.2.3 Development Safety Update Reports

Development Safety Update Reports (DSURs) will be prepared for the trial. The reports will be written by the CI and CRUK CTU on behalf of the Sponsor. The CRUK CTU will submit DSURs to the Regulatory Authorities, REC, trial sites, Sponsor and Fuji Film Pharmaceuticals who are supplying the IMP on the anniversary of obtaining the UK Clinical Trial Authorisation.

6.2.4 Auxiliary Medicinal Products (AMPs)

There are no AMPs in this protocol.

6.2.5 Reference Safety Information

The section of the IB titled Reference Safety Information (RSI) supplied by Fuji Film Pharmaceuticals for favipiravir will act as the RSI for the trial. Fuji Film Pharmaceuticals is responsible for supplying the Sponsor with updated IBs within 14 days of UK regulatory approval. However, only the RSI section of the IB with current regulatory approval to act as the RSI for the GETAFIX trial, will be used to assess expectedness (with the proviso that the RSI must remain consistent for each DSUR reporting period). Investigators will be supplied with IBs and updated IBs when the Sponsor and CI conclude the update will assist with the clinical management of trial patients.

6.2.6 Changes to the RSI or Risk-Benefit Assessment

If changes to the RSI include the addition of new reactions now considered expected, an update that impacts on patient safety or if the risk-benefit assessment or clinical management of trial participants is affected by an update to the RSI, then the RSI will not change for the duration of the DSUR reporting period however the updated IB will:

- Circulated to trial sites
- A front sheet document for use when the risk benefit or clinical management has changed, will accompany the reference IB and provide guidance on the current RSI and IB be referred to for the clinical management of trial patients

- If patient safety/or the risk-benefit assessment has changed and/or new expected reactions have been added, then approval to use the updated IB as the RSI, will be made by submitting a substantial amendment to the Regulatory Authority.
- If new reactions are added to the IB as "expected", implementing the updated IB for assessing SARs will not occur until regulatory approval is received for the updated IB and once the new DSUR reporting period starts

Changes that impact on patient safety or alter the risk-benefit assessment may require changes to the trial documentation such as the patient information sheet. The CI on behalf of the sponsor will identify any such required changes. The CTU will seek approval of the changes, as required by the clinical trial regulations.

6.2.7 Requirements for DSUR Reporting and the Maintenance of RSI in the Follow-Up Period

DSURs will continue to be submitted and the RSI maintained until the last patient has attended for their last follow-up visit.

7 STATISTICS AND DATA ANALYSIS

7.1 Trial Design and Sample Size

The study is designed as an open-label randomised (1:1) phase II/III study which allows for early stopping for lack of efficacy or clear superiority in the experimental arm.

The study is powered to detect an improvement corresponding to an odds ratio of 1.95 in the cumulative odds of the WHO COVID 10-point ordinal severity scale assessed at day 15. This is equivalent to the probability of a patient having a better outcome at this time if allocated to experimental as compared to control of 66% (50% under null hypothesis). The following table illustrates that improvement:-

	**WHO COVID 10-point ordinal severity scale**	**Assumed percentages on control arm at day 15***	**Percentages on experimental arm at day 15 with odds ratio=1.95**
Death	Death	11%	6.0%
Hospitalized; severe disease	Mechanical ventilation pO2/FIO2<150 and vasopressors, dialysis or ECMO	5%	2.9%
	Mechanical ventilation pO2/FIO2>150 (SpO2/FIO2> 200) or vasopressors	7%	4.4%

	ICU - intubation & mechanical ventilation, pO2/FIO2>150 or SpO2/FIO2> 200	5%	3.3%
Hospitalized; Mild disease	Hospitalized oxygen by NIV or High flow	13%	9.6%
	Hospitalized on supplemental oxygen	13%	11.3%
	Hospitalized not on supplemental oxygen	12%	12.3%
Ambulatory	Symptomatic – assistance needed	2%	2.3%
	Symptomatic – independent	16%	21.8%
	Asymptomatic – viral DNA detected	16%	27.0%

*Abstracted from figure 6 in ISARIC COVID-19 Report: 02 April 2020

To detect this magnitude of difference with 90% power at the 5% 2-sided level of statistical significance requires 302 patients (151 per arm). This allows for the possibility of early stopping at two interim analyses after primary outcome data is available for 128 and 202 patients. The interim analyses are detailed in 7.3.

7.1.1 Analysis Plan

Analyses to account for any non-trial treatment for COVID-19 will be investigated prior to any analyses being undertaken. The decision regarding whether adjustments are required may be based on a blinded assessment of the number of patients who receive non-trial treatment for COVID19 prior to each outcome time-point being reached. Analyses will be used to remove confounding from non-trial COVID19 treatment, and may include inverse probability weighting.

7.1.2 Primary Efficacy Analysis

The primary analysis will use cumulative odds ordinal regression with proportional odds. The model terms will be study arm and the minimisation factors used for randomisation. The odds of a patient having a better outcome if allocated to the experimental arm will be estimated along with the associated 95% confidence interval. A Forest plot illustrating treatment effect odds ratios by minimisation factor categories will also be provided. . A test for interaction will be conducted to assess whether the effect of treatment arm depends on the other clinical factors used in the minimisation algorithm.

It will not be possible to distinguish between 0 and 1 on the WHO COVID 10-point ordinal severity scale so these will be combined in analyses.

7.1.3 Secondary Efficacy Analysis

Proportion of patients meeting level 7 or above on the WHO COVID 10 point ordinal severity scale or dead by day 29; Overall survival over the 60 days; Viral clearance on or before d8 of treatment (inpatients)

These end-points will be tabulated and examined using logistic regression incorporating terms for study arm and the minimisation factors used for randomisation.

Duration of pyrexia (inpatients only)

This will be summarised as time from developing pyrexia (taken as the date of admission to hospital if present at randomisation) using a Cumulative Incidence Function plot and analysed using Fine and Gray regression incorporating terms for study arm and the minimisation factors used for randomisation. (Gray, 1988, a class of k-sample tests for comparing the cumulative incidence of a competing risk. Annals of Statistics 1988; 16:1141-1154)(Fine, 1999, A proportional hazards model for the subdistribution of a competing risk. Journal of the American Statistical Association 1999; 94: 496-509). Patients who die during the course of the 60 day study period, prior to resolution of pyrexia, will be considered as having had a competing risk at the date of death. Participants who never develop pyrexia will not be included in this analysis.

WHO COVID 10-point ordinal severity scale at day 8, 29, 60

This will be analysed using the same technique as the primary end-point.

7.1.4 Exploratory Endpoint Analysis

Duration of hospitalization (inpatients only)

This will be summarised using a Cumulative Incidence Function plot and analysed using Fine and Gray regression incorporating terms for study arm and the minimisation factors used for randomisation. (Gray, 1988, a class of k-sample tests for comparing the cumulative incidence of a competing risk. Annals of Statistics 1988; 16:1141-1154)(Fine, 1999, A proportional hazards model for the subdistribution of a competing risk. Journal of the American Statistical Association 1999; 94:

496-509). Patients who die during the course of the 60 day study period (if still in hospital) will be considered as having had a competing risk at the date of death.

Change in viral load

Viral load will be summarised and compared between treatment groups using an area under curve (AUC) approach standardised for the period on study and using the baseline value as a covariate.

Viral clearance on or before d8 of treatment (outpatients)

This will be tabulated and examined using logistic regression incorporating terms for study arm and the minimisation factors used for randomisation, if the number of patients is sufficient.

Evaluate the relationship between post COVID19 health and psycho-social consequences and the clinical outcomes of the study

This will be defined prior to analysis, and may include combined analyses with other studies, such as the ISARIC study.

7.1.5 Safety

The worst toxicity grades (including AEs of special interest) experienced during active treatment will be compared between the study arms using the Mann-Whitney U test. P-values will be interpreted in the context of the multiple tests performed. This analysis with be restricted to events that occur in more than 10% of patients in either arm.

All Serious Adverse Events (including SARs and SUSARs) will be tabulated.

7.2 Interim Analysis

The stopping rules for the interim analyses are based on group sequential asymmetric boundaries using Hwang-Shih-DeCani spending functions with gamma=-2.6 (lower boundary) and gamma=-9.5 (upper boundary). The upper boundary is binding for early stopping; the lower boundary is non-binding and the Data Monitoring Committee may take into account the effect on secondary end-points in reaching their decision on whether the study should proceed or not.

		Lower boundary			Upper boundary		
Analysis	N	Z	Nominal p	Beta-Spend	Z	Nominal p	Alpha -Spend

Interim 1	128	0.00	0.5020	0.0160	3.72	0.0001	0.0001
Interim 2	202	0.84	0.7992	0.0218	3.08	0.0010	0.0010
Final	302	1.96	0.9751	0.0622	1.96	0.0249	0.0239
Total				*0.1000*			*0.0250*

At the first interim analyses the distribution of the primary end-point in the control arm will be reviewed by the Trials Steering Committee (TSC) to determine how well it conforms to the initial assumptions and whether or not any modifications may be required to the sample size calculations. This review is required because of limited experience with the primary end-point in this patient population. The TSC has no access to outcome data on the experimental arm.

8 TRIAL CLOSURE

The end of trial is defined when the Trial Steering Committee agrees that one or more of the following situations apply:

- Six months after the last patient is recruited
- Final follow-up visit of the last patient
- The stated objectives of the trial are achieved.

It is the responsibility of the Sponsors to inform the Medicines and Healthcare products Regulations Agency (MHRA) and the Main Research Ethics Committee (REC) within 90 days of the 'end of the trial' that the trial has closed. In cases of early termination of the trial (for example, due to toxicity) or a temporary halt by the Sponsors, the Sponsors will notify the MHRA and the Main REC within 15 days of the decision and a detailed, written explanation for the termination/halt will be given.

Regardless of the reason for termination, all data available for the patient at the time of discontinuation of follow-up must be recorded in the eCRF. All reasons for discontinuation of treatment must be documented.

In terminating the trial, the Sponsors and the Investigators must ensure that adequate consideration is given to the protection of the patient's interest.

8.1 End of Trial Notification/Declaration of the End of a Study Form

End of trial notification will be submitted to the competent authority and ethics committee within 90 days using the 'Declaration of the end of a clinical trial' form that can be found on the EudraCT website. However if the trial is terminated either (1) before the date for the conclusion of the trial specified in the protocol for that trial or (2) before the number of events required by the trial has occurred, the competent authority and ethics committee will be notified in writing of the termination of the trial within 15 days of the date of termination with a clear explanation of reasons and details of follow-up measures, if any, taken for safety reasons.

8.2 Clinical Trial Summary Report

The clinical trial summary report should be submitted to the competent authority within one year of submitting the end of trial notification. The content and format of the report should follow guidelines published on the MHRA website. The CI in association with CRUK CTU is responsible for compiling and submitting the final report to both sponsors, MHRA and the REC.

8.3 Temporary Halt of a trial

If recruitment to the trial needs to be temporarily halted for reasons not specified in the protocol (e.g. unexpected availability of trial IMP) the Sponsor will inform the MHRA and/or REC immediately and at the latest within 15 days from when the trial is temporarily halted. This includes trials where the stoppage was not envisaged in the approved protocol and where there is an intention to resume it. It does not include trials where recruitment may be temporarily halted for logistical reasons such as trial team availability. The notification will be made as a substantial amendment and will clearly state what activities have been halted and the reasons for this. To restart a trial that has been temporarily halted the Sponsor will make a request as a substantial amendment providing evidence that it is safe to restart the trial. If the Sponsor decides not to recommence the trial the MHRA and REC will be notified in writing within 15 days of the decision, using the end-of-trial declaration form.

8.4 Early Termination of a Trial

In the case of early termination the Sponsor will notify the end of a trial to the MHRA and/or REC immediately and at the latest within 15 days after the trial is halted, explaining the reasons and describing the follow-up measures, if any, to be taken for safety reasons. This does not include trials that complete early because full recruitment has been achieved.

9 DATA HANDLING

9.1 CRFs

The CRFs for this trial will be completed using the electronic remote data capture (eRDC) system, MACRO®. Prior to recruitment beginning at each site, the MACRO® User Guide will be sent to sites.

It is the responsibility of the Principal Investigator to ensure eCRFs are completed in a timely manner (within 4-6 weeks of the study visit) and to review and approve all data captured on the eCRF. **Please ensure that all data submitted on eCRFs are verifiable in the source documentation or that any discrepancies are recorded and explained.**

In addition to completing the MACRO® database there will be some paper CRFs, the randomisation/registration form should be completed on the paper form prior to faxing or calling CRUK CTU. SAE forms are also completed on paper.

Please review to the data completion guideline document in the ISF. Please also note that some study forms must be signed by the PI or another clinician delegated to do so on the delegation log. These forms will be defined in the completion guidelines.

Other essential documents, including source data, consent forms, and regulatory documentation, will be archived by, or for the Investigator, in an appropriate archive facility in line with current regulatory requirements and made available for monitoring, audit and regulatory inspection as required.

9.2 Central Review of Data

CRUK CTU will regularly review the data for compliance with the protocol, and for inconsistent or missing data. Should any missing data or data anomalies be found within the eCRFs upon CTU review, queries will be generated within the MACRO® study database for the site to access and resolve. Sites are expected to review and respond to queries within the database in a timely manner

(within 2-3 weeks). Any issues identified at sites in relation to poor data/slow response to data queries will be managed as per the data escalation process below.

9.3 Data Escalation Processes

Where issues with data return/quality/response to requests are identified at sites, the following process will be followed:

Step 1: E-mail letter to site main contact and copy in site PI
Step 2: E-mail letter direct to site PI and copy in site main contact
Step 3 E-mail letter to Network Coordinator and copy in site PI and main contact
Step 4: Discuss suspension of recruitment at site until data issues resolved

9.4 Record Retention and archiving

Archiving of the trial essential documents should be performed by both the participating trial site and Sponsor/CRUK CTU.

Participating sites are responsible for archiving their trial related documentation and should follow the requirements of their R&D Office in conjunction with advice from the CRUK CTU and Sponsor regarding the duration of document retention. Sites should not archive their trial documentation until they have been instructed by the CRUK CTU or Sponsor that they are able to do so. Where possible, at the time of archiving, sites will be notified of the archiving retention period. If this is not confirmed at the time of archiving, sites should not destroy archived documentation until authorisation is given from the Sponsor.

The Sponsor and CRUK CTU will be responsible for archiving the Trial Master File (TMF) and all other essential trial documentation that is not held at participating trial sites as per their applicable SOPs.

In the event that a patient's care is transferred to another hospital a Patient Transfer Form must be completed by the original recruiting site (or the current site responsible for the patient) to request that the transfer is performed within the CTU and MACRO® system. The original recruiting site will be recognised with the recruitment of the patient. The original (or current) site will be responsible for ensuring all is up-to-date prior to the transfer of the patient on the MACRO® system. Once the

transfer has been processed, the new site will be responsible for returning all outstanding trial documents from that point onwards including any outstanding data prior to the date of transfer.

9.5 PK/PD data analysis

Pharmacokinetic and Pharmacodynamic analyses will be performed by The Pharmacy department, University of Strathclyde, and the University of Glasgow Translational Pharmacology Laboratory respectively.

10 TRIAL MANAGEMENT

The Clinical Trial Project Manager will be responsible for the ongoing coordination and management of the trial. The Sponsors are responsible for all duties relating to pharmacovigilance in accordance with section 7. Before the trial can be initiated, the prerequisites for conducting the trial must be clarified and the organisational preparations made with the trial centre. The Sponsor must be informed immediately of any change in the personnel involved in the conduct of the trial.
During the trial, the Sponsor's Clinical Trial Monitor (CTM) is responsible for monitoring data quality in accordance with Sponsor's standard operating procedures (SOPs).
All clinical data will be presented at the end of the trial on final data listings. The CI and CRUK-CTU Glasgow will prepare a clinical study report based on the final data listings. The report will be submitted to the Investigator(s) and NHS Greater Glasgow and Clyde / University of Glasgow (sponsors) for review and confirmation it accurately represents the data collected during the course of the trial. A summary of the final clinical report must be provided by the CRUK-CTU Glasgow to the MHRA and to the Research Ethics Committee.

It is the responsibility of the CRUK-CTU Glasgow to inform the Main REC within 90 days of the 'end of the trial' that the trial has closed.

10.1 Trial Start Up

Sites wishing to participate in the trial should contact CRUK CTU. A PI must lead the trial at each site and they will be responsible for providing CRUK CTU with all core documentation. Protocol training will be given to sites via initiation slides that will be provided to sites prior to the trial opening at that site. Once all the documentation is received at CRUK CTU Glasgow an initiation call

will be performed and after this the site will be contacted by email or fax when they are activated and are able to recruit patients to the trial.

10.2 Core Documents

The Core Documents include:

- Local R&D approval/local capacity and capability approval/HRA approval
- Signed Clinical Trial Agreement
- Delegation and Study Specific Training Log
- CV and GCP for PI and lead pharmacist
- Lab accreditation certificates
- PIS/ICF/GP letter on local headed paper
- Initiation acknowledgements
- Pharmacy assessment forms
- Site initiation/accreditation checklist
- MACRO® User Request Forms

10.3 Management of protocol deviations and violations

Deviations

Organisations must notify the co-Sponsor (via CRUK CTU) of all deviations from the protocol or GCP immediately. The co-Sponsor requires a report on the incident(s) and a deviation form will be provided to site for completion. This should be completed by site as soon as possible and returned to the PM or CTM. If site staff are unsure whether a certain occurrence constitutes a deviation from the protocol or GCP, the CRUK CTU trial team and Co-Sponsor can be contacted immediately to discuss. The Co-Sponsor will assess all incidents with respect to the criteria of a "serious breach".

Serious Breach

Events that match the criteria of a "serious breach" will be reported to the MHRA and REC within 7 days of the matter coming to the attention of the (co-) Sponsor. The report should include details of when the breach occurred, the location, who was involved, the outcome and any information given to the participants. The MHRA should also be informed of any further corrective or preventative action the (co-)Sponsor plan to take.

In addition to the definition of a serious breach in GCP, systematic or persistent violation by a site of GCP and/or the protocol, including failure to report SAEs occurring on trial within the specified timeframe, may be deemed a serious breach.

Sites must have written procedures for notifying the Sponsors of serious breaches (MHRA Guidance on the Notification of Serious Breaches, 2009).

The Sponsors and CRUK CTU will use an organisation's history of non-compliance to make decisions on future collaborations.

10.4 Trial Management Group (TMG)

The trial will be coordinated from CRUK CTU by the TMG. The TMG normally includes those individuals responsible for the day-to-day management of the trial. Members of the TMG include the Chief Investigator, Co-Investigators, Project Manager, Trial Statistician, Clinical Trial Co-ordinator, Clinical Trial Monitor, Pharmacovigilance Monitor, and Sponsor Pharmacist. The role of the group is to monitor all aspects of the conduct and progress of the trial, ensure that the protocol is adhered to and take appropriate action to safeguard participants and the quality of the trial itself.

10.5 COVID-19 Umbrella Trial Steering Committee (COVID-19 UTSC)

The role of the COVID-19 UTSC is to provide overall supervision of the trial and ensure that it is being conducted in accordance with the principles of GCP and the relevant regulations. The TSC should agree any significant protocol amendments, provide advice to the investigators on all aspects of the trial and have members who are independent of the investigators, in particular an independent chairperson. Decisions about continuation or termination of the trial or substantial amendments to the protocol are usually the responsibility of the TSC.

10.6 COVID-19 Umbrella Independent Data Monitoring Committee (COVID-19 UIDMC)

The role of the COVID-19 UIDMC is to review the accruing trial data and to assess whether they are any safety or efficacy issues that should be brought to participants' attention or any reasons for the trial not to continue. In particular they will review the data at the prespecified interim analyses. The IDMC will be independent of both the investigators and will be the only body that may have access to unblinded data during the course of the trial. It will make recommendations to the TSC.

11 REGULATORY ISSUES

11.1 Clinical Trials Authorisation (CTA)

On behalf of the trial Sponsor, CRUK CTU Glasgow will apply to the MHRA for a Clinical Trials Authorisation (CTA) to conduct the trial and will also be responsible for maintenance of the CTA.

11.2 Ethics Approval

The trial will be carried out in accordance with the World Medical Association Declaration of Helsinki (1964) and its revisions (Tokyo [1975], Venice [1983], Hong Kong [1989], South Africa [1996] and Edinburgh [2000]).

Favourable ethical opinion will be sought from a Research Ethics Committee (REC) before patients are entered onto this clinical trial. The CI will be responsible for updating the ethics committee of any new information related to the trial.

11.3 Consent

Consent to enter the trial must be sought from each participant only after full explanation has been given, an information sheet offered and time allowed for consideration. Signed participant consent must be obtained, the consent forms should also be signed by the person carrying out the consent procedure at site, who must be detailed on the study specific delegation and training log as having authorisation. For patients who have capacity to understand and give consent, but are unable to read the consent form or sign their name, witness consent may be given. The PI is responsible for ensuring if the taking of consent is delegated to a designee, the designee is suitably qualified by training or experience to take informed consent.

The right of the participant to refuse to participate without giving reasons must be respected. After the participant has entered the trial the clinician remains free to give alternative treatment to that specified in the protocol at any stage if he/she feels it is in the best interests of the participant, but the reasons for doing so must be recorded. In these cases the participants remain within the trial for the purposes of follow-up and data analysis. All participants are free to withdraw at any time from the protocol treatment without giving reasons and without prejudicing further treatment.

An original completed consent form must be retained at each site in the appropriate section of the Investigator Site File, and a photocopy placed in the patient's medical records. All patients must be given either an original or a copy (as per local site practice) of the signed patient information sheet and consent form for their records. Consent forms must be retained on site and not submitted to the CRUK CTU Glasgow.

In the event that new patient information sheets/consent forms are produced throughout the duration of the trial, it maybe that patients already participating in the trial should be re-consented to the updated version of the patient information sheet. However, if the principal investigator decides that this is not in the best interests of the patient re-consent is not required. Decisions not to re-consent patients must be documented in the patient's medical records.

11.4 Confidentiality

All information collected during the course of the trial will be kept strictly confidential.

Information will be held securely on paper and electronically at the CRUK CTU. The CRUK CTU will comply with all aspects of the 1998 Data Protection Act and operationally this will include:

- Consent from participants to record personal details including initials, date of birth, CHI/NHS number, hospital number, GP name and address.
- Appropriate storage, restricted access and disposal arrangements for patient's personal and clinical details
- Consent from participants for access to their medical records by responsible individuals from the research staff or from regulatory authorities, where it is relevant to trial participation
- Consent from participants for the data collected for the trial to be used to evaluate safety and develop new research.
- Where central monitoring of source documents by CRUK CTU (or copies of source documents) are required (such as scans or local blood results), the patient's name must be obliterated by site before sending.
- Where anonymisation of documentation is required, sites are responsible for ensuring only the instructed identifiers are present before sending to CRUK CTU.

If a participant withdraws consent from further trial treatment and / or further collection of data their samples will remain on file and will be included in the final trial analysis unless they specifically withdraw consent for this.

11.5 Liability, Indemnity and Insurance

No special insurance is in place for patients in this trial other than standard NHS liability insurance providing indemnity against clinical negligence. This does not provide cover for non-negligence e.g. harm caused by an unexpected side effect of participating in a trial. The sponsors have responsibility for ensuring that financial cover for damages or compensation arising from no fault harm is available to patients, where applicable. The co-sponsor, University of Glasgow, maintains clinical trials insurance. Cover for this clinical trial has been agreed under the current policy.

The Hospital Trust/Health Board at each participating site is responsible for the following:

1. Acts and omissions of its own staff and others engaged by it, including the Clinical Trials Unit and PI;
2. Ensuring the appropriate insurance administered by the National Health Service Litigation Authority is in place;
3. Ensuring any non-NHS employees involved in the clinical trial have Honorary Contracts with the Trust/Board to cover access to patients and liability arrangements.

These responsibilities are outlined and agreed within the Clinical Trial Agreement.

11.6 Sponsor(s)

NHS Greater Glasgow and Clyde and The University of Glasgow will act as co-sponsors for this trial. Delegated activities will be assigned to the CRUK CTU and NHS Trusts/Boards taking part in this trial. Details of responsibilities will be outlined in the clinical trial agreement that should be signed prior to site initiation.

11.7 Funding

Funding for the study has been provided by the Chief Scientist Office.

11.8 Protocol Amendments

Any change to the trial protocol will require an amendment. Any proposed, non-administrative, protocol amendments will be initiated by the CI following discussion with the TMG and any required amendment forms will be submitted to the regulatory authority, ethics committee and sponsor(s). The CI and the TMG will liaise with trial sponsor(s) to determine whether an amendment is non-

substantial or substantial. All amended versions of the protocol will be signed by the CI and sponsor(s) representative. Before the amended protocol can be implemented favourable approval must be sought from the original reviewing REC, trial Sponsors, MHRA and participating site R&D offices.

11.9 Allocation of Trial Responsibilities

11.9.1.1 Co-Sponsor Responsibilities (NHS GG&C/University of Glasgow)

The Sponsor is responsible for confirming there are proper arrangements for the initiation and management of the trial. Any Sponsor's responsibilities that have been delegated to the CI will be documented within the 'Responsibilities delegated to the Chief Investigator' form. The duties will be performed via the CRUK CTU as the co-ordinating centre for the trial.

11.9.2 Chief Investigator (CI)

The CI is directly responsible for:

- Ensuring the protocol and any amendments are in place.
- Clinical oversight of the safety of patients participating in the trial, including the ongoing review of the risk/benefit.
- For review of SAEs and determination if SAEs meet the criteria for a SUSAR within 24 hours.
- Providing advice and recommendations on medical issues that arise involving the management of the patients on the trial.

At the outset of the trial development period, the CI will sign the CRUK CTU Memorandum of Understanding (MoU) document which details the key responsibilities of the CI and CRUK CTU, where applicable giving indicative timelines for completion. In addition, the CI will sign the (co-) Sponsor Responsibilities Agreement.

From the perspective of the Co-Sponsor and for regulatory/ethics purposes, the CI for the trial will be Prof Rob Jones.

11.9.3 CRUK Clinical Trials Unit (CTU)

The CRUK CTU delivers the overall management of the clinical trial. This includes, but is not limited to, all regulatory submissions (ethics, HRA, R&D and CTA) and any amendments, all administration relating to the submissions and any amendments, circulation of all correspondence to participating sites, data management, monitoring of data quality and safety, ongoing communication with

participating sites, management of SAE/SUSAR reporting, and where applicable the management of any financial arrangements.

11.9.4 Participating Site

The Participating Site is solely responsible for the management of the trial within their site. This includes ensuring local management approval has been given, ensuring the trial is conducted according to GCP requirements, and ensuring the appropriate insurance or indemnity is in place. The Participating Site is also responsible for arranging access for on-site monitoring and auditing as identified in the trial protocol and also for regulatory inspections.

11.9.5 Principal Investigator (PI)

The PI is responsible for:

- The delegation of trial activities within their site and ensuring all personnel are adequately trained and qualified to carry out their responsibilities.
- Providing evidence of GCP training (usually a certificate) or undergo the required GCP training.
- The safety and wellbeing of trial patients,
- Reporting any deviations from the protocol to CRUK CTU Glasgow
- Reporting any SAEs or safety issues within 24 hours of becoming aware of the event, including using medical judgement in assigning seriousness, causality and expectedness using the RSI approved for the study.

Full details of the responsibilities of the PI are outlined in the Clinical Trial Agreement. Two original copies of this will be held – one with the Sponsor(s) and the other at the participating site. A photocopy of the signed agreement will also be held at the coordinating trial office.

11.10 Non-medical Prescribing (if appropriate)

Non-medical prescribing may be permitted by the sponsor, if it is local practice for these staff to undertake this duty. These staff must be delegated the duty of prescribing within the delegation and study specific training log, by the PI, hold an accepted prescribing qualification and full, current GCP training certificate.

12 QUALITY ASSURANCE

12.1 Audits and Inspections

Trial Investigators must permit trial related monitoring, audits, REC review and regulatory inspections as required, by providing direct access to source data, CRFs and other documents (patients' medical records, investigator site file, and other pertinent data).

The trial may be subject to inspection and audit by <Sponsor> as Sponsor(s), the CRUK CTU and other regulatory bodies, i.e. the MHRA, to ensure adherence to GCP. If an inspection is scheduled at any participating site, the site must notify the Sponsor(s) at the earliest opportunity.

It is the sponsor's responsibility to inform the investigator(s) of all intended audits and regulatory inspections involving the participating site. It is the investigator's responsibility to ensure appropriate resources at site and that the inspector(s) have access to all source data.

12.2 On Site and Telephone Monitoring

Participating study sites will be monitored remotely by the CRUK CTU on behalf of the Sponsor(s) by both telephone and remote review of data. The CRUK CTU reserves the right to undertake for-cause monitoring should this be considered necessary at any stage. The level of monitoring have been agreed in advance between the Project Manager and Clinical Trial Monitor with input from the Quality Assurance Manager and Sponsor via the trial risk assessment. The PI and Site Pharmacy staff will allow the Clinical Trial Monitor remote access to relevant trial data as requested. Investigators and site staff will be notified in advance about forthcoming monitoring.

12.3 Protocol non-compliance

Protocol non-compliances must be reported by the site study team to the CRUK CTU as soon as they are identified. Non-compliances may also be identified by the Clinical Trial Monitor, and the site staff and CRUK CTU staff will work together to complete a protocol deviation form and put corrective and preventive actions in place to avoid repeated non-compliance. Where the deviation is of a more serious nature, the Sponsor may be required to report a serious breach of protocol or Good Clinical Practice to the MHRA. The Sponsor reserves the right to suspend recruitment at a site until an

investigation has taken place and corrective and preventive measures have been put in place to ensure future patient safety and/or data integrity.

13 PUBLICATION POLICY

The GETAFIX TMG is responsible for approving the content and dissemination of all publications, abstracts and presentations arising from the trial and for assuring the confidentiality and integrity of the trial. It will provide collaborators the International Committee of Medical Journal Editors (ICMJE)(http://www.icmje.org/recommendations/browse/roles-and-responsibilities/defining-the-role-of-authors-and-contributors.html#two) used to ensure all those who have contributed to the study are appropriately acknowledged.

No site or individual will publish data without prior approval of the TMG.

The data arising from GETAFIX will belong to the trial Sponsors (NHS Greater Glasgow and Clyde and University of Glasgow). The TMG shall act as custodian of this data.

14 REFERENCES

1. Bai CQ, Mu JS, Kargbo D, et al. Clinical and Virological Characteristics of Ebola Virus Disease Patients Treated With Favipiravir (T-705)-Sierra Leone, 2014. *Clinical Infectious Diseases* 2016; **63**(10): 1288-94.
2. Sissoko D, Laouenan C, Folkesson E, et al. Experimental Treatment with Favipiravir for Ebola Virus Disease (the JIKI Trial): A Historically Controlled, Single-Arm Proof-of-Concept Trial in Guinea. *Plos Medicine* 2016; **13**(3).
3. Organisation WH. WHO COVID19 Situation Report. 13th April 2020. https://www.who.int/docs/default-source/coronaviruse/situation-reports/20200412-sitrep-83-covid-19.pdf?sfvrsn=697ce98d_4 .
4. Wang M, Cao R, Zhang L, et al. Remdesivir and chloroquine effectively inhibit the recently emerged novel coronavirus (2019-nCoV) in vitro. *Cell research* 2020.
5. Cai Q, Yang M, Liu D, et al. Experimental Treatment with Favipiravir for COVID-19: An Open-Label Control Study. *Engineering* 2020.
6. Organisation WH. Therapeutics Landscape WHO. 2020. https://www.who.int/blueprint/priority-diseases/key-action/Table_of_therapeutics_Appendix_17022020.pdf?ua=1.
7. Smee DF, Hurst BL, Egawa H, Takahashi K, Kadota T, Furuta Y. Intracellular metabolism of favipiravir (T-705) in uninfected and influenza A (H5N1) virus-infected cells. *Journal of Antimicrobial Chemotherapy* 2009; **64**(4): 741-6.
8. Furuta Y, Takahashi K, Shiraki K, et al. T-705 (favipiravir) and related compounds: Novel broad-spectrum inhibitors of RNA viral infections. *Antiviral Research* 2009; **82**(3): 95-102.
9. Mendenhall M, Russell A, Smee DF, et al. Effective Oral Favipiravir (T-705) Therapy Initiated after the Onset of Clinical Disease in a Model of Arenavirus Hemorrhagic Fever. *Plos Neglected Tropical Diseases* 2011; **5**(10).

10. Madelain V, Guedj J, Mentre F, et al. Favipiravir Pharmacokinetics in Nonhuman Primates and Insights for Future Efficacy Studies of Hemorrhagic Fever Viruses. *Antimicrobial Agents and Chemotherapy* 2017; **61**(1).
11. Wang M, Cao R, Zhang L, et al. Remdesivir and chloroquine effectively inhibit the recently emerged novel coronavirus (2019-nCoV) in vitro. *Cell Res* 2020; **30**(3): 269-71.
12. Chen C, Huang J, Cheng Z, et al. Favipiravir versus Arbidol for COVID-19: A Randomized Clinical Trial. 2020.
13. Chinese Clinical Trial Registry. Clinical study for safety and efficacy of Favipiravir in the treatment of novel coronavirus pneumonia (COVID-19). 2020. http://www.chictr.org.cn/showprojen.aspx?proj=49042 (accessed 25th April 2020.
14. .gov. Efficacy and Safety of Favipiravir in Management of COVID-19 (FAV-001). 2020. https://clinicaltrials.gov/ct2/show/NCT04349241 (accessed 25th April 2020.
15. Chinese Clinical Trial Registry. A Randomized Controlled Trial for Favipiravir Tablets Combine With Chloroquine Phosphate in the Treatment of Novel Coronavirus Pneumonia (COVID-19). 2020. http://www.chictr.org.cn/showprojen.aspx?proj=51329 (accessed 25th April 2020.
16. Chinese Clinical Trial Registry. Favipiravir Combined With Tocilizumab in the Treatment of novel coronavirus pneumonia (COVID-19) - A Multicenter, Randomized, Controlled Trial. 2020. http://www.chictr.org.cn/showprojen.aspx?proj=51126 (accessed 25th April 2020.
17. Chinese Clinical Trial Registry. Randomized controlled trial for safety and efficacy of Favipiravir in the treatment of novel coronavirus pneumonia (COVID-19) with poorly responsive ritonavir/ritonavir. 2020. http://www.chictr.org.cn/showprojen.aspx?proj=49988 (accessed 25th April 2020.
18. Chinese Clinical Trial Registry. Randomized, open-label, controlled trial for evaluating of the efficacy and safety of Baloxavir Marboxil, Favipiravir, and Lopinavir-Ritonavir in the treatment of novel coronavirus pneumonia (COVID-19) patients. 2020. http://www.chictr.org.cn/showprojen.aspx?proj=49015 (accessed 25th April 2020.
19. Chinese Clinical Trial Registry. A randomized controlled trial for the efficacy and safety of Baloxavir Marboxil, Favipiravir tablets in novel coronavirus pneumonia (COVID-19) patients who are still positive on virus detection under the current antiviral therapy. 2020. http://www.chictr.org.cn/showprojen.aspx?proj=49013 (accessed 25th April 2020.
20. Hochberg Y. A sharper Bonferroni procedure for multiple tests of significance. *Biometrika* 1988; **75**(4): 800-2.
21. Qian W, Parmar MK, Sambrook RJ, Fayers PM, Girling DJ, Stephens RJ. Analysis of messy longitudinal data from a randomized clinical trial. MRC Lung Cancer Working Party. *Statistics in medicine* 2000; **19**(19): 2657-74.

15 APPENDICES

Appendix 1 : WHO 10-point COVID Severity Scale

Patient State	Descriptor	Score
Uninfected	Uninfected; no viral RNA detected	0
Ambulatory	Asymptomatic; viral RNA detected	1
	Symptomatic; Independent	2
	Symptomatic; Assistance needed	3
Hospitalized: Mild disease	Hospitalized; no oxygen therapy	4
	Hospitalized; oxygen by mask or nasal prongs	5
Hospitalized: Severe disease	Hospitalized; Oxygen by NIV or High flow	6
	Intubation & Mechanical ventilation, $pO_2/FIO_2 \geq 150$ or $SpO_2/FIO_2 \geq 200$	7
	Mechanical ventilation $pO_2/FIO_2 < 150$ ($SpO_2/FIO_2 < 200$) **or** vasopressors	8
	Mechanical ventilation $pO_2/FIO_2 < 150$ **and** vasopressors, dialysis, or ECMO	9
Death	Dead	10

Appendix 2: World Health Organisation (WHO) performance score

WHO Performance Status Grade	Description
0	Fully active, able to carry on all pre-disease performance without restriction
1	Restricted in physically strenuous activity but ambulatory and able to carry out work of a light or sedentary nature, e.g., light house work, office work
2	Ambulatory and capable of all self-care but unable to carry out any work activities. Up and about more than 50% of waking hours
3	Capable of only limited self-care, confined to bed or chair more than 50% of waking hours
4	Completely disabled. Cannot carry on any self-care. Totally confined to bed or chair
5	Dead

Appendix 3: Questionnaire

The ISARIC4C COVID-19 Health and Wellbeing Initial Follow Up Surveys (Initial and Follow-up) will be provided as separate documents

Appendix 4 Child Pugh Classification

Child Pugh classification

		Score	
**Clinical/laboratory findings**	**1**	**2**	**3**
**Encephalopathy**	None	Mild (grade 1-2)	Severe (grade 3-4)
**Ascites**	None	Mild/Slight	Moderate/Large
**Bilirubin (micromol/l)**	<34	34-51	>51
**Albumin (g/l)**	≥35	28-35	<28
**Prothrombin time prolongation (secs)**	<4	4-6	>6
**or international normalised ratio (INR)**	<1.3	1.3 – 1.5	>1.5

Chronic liver disease is classified into Child-Pugh class A to C, employing the total score from the above table.

Total Points	Child-Pugh class
5-6	A
7-9	B
10-15	C

Appendix 5 PK Sampling Schedule

Inpatients

PK Sample to be taken at:

Day 1: pre and post dose (0.5 hours)
Day 3: pre and post dose (0.5 hours)
Day 5: pre and post dose (0.5 hours)
Day 8: Pre dose
Day 10: Pre dose

In addition, 16 volunteers (8 male, 8 female) will have more frequent sampling for PK on days 1 and 3 after either dose that day. Hours post dose: 0.5, 1, 2, 7, 12

Outpatients

PK samples to be taken pre dose (screening or day 1), and post dose day 1 (0.5 hours)
