# Supplementary material for: Glasgow Early Treatment Arm Favirpiravir (GETAFIX) for adults with early stage COVID-19: A structured summary of a study protocol for a randomised controlled trial
Source: Trials. 2020 Nov 19;21:935. doi: 10.1186/s13063-020-04891-1 (PMC7675389; doi:10.1186/s13063-020-04891-1)
Supplement: Supplementary file 2 — Additional file 2. [file 13063_2020_4891_MOESM2_ESM.docx]

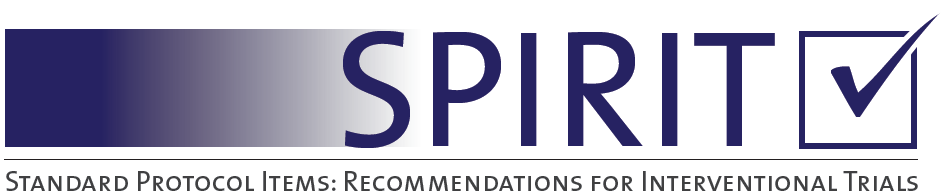


| **Section/item** | **Item No** | **Description** | | | | | | | | |
| --- | --- | --- | --- | --- | --- | --- | --- | --- | --- | --- |
| **Administrative information** | | | | | | | | | | |
| Title | 1 | Glasgow Early Treatment Arm Favipiravir (GETAFIX): A randomised controlled study of favipiravir as an early treatment arm in COVID-19 patients | | | | | | | | |
| Trial registration | 2a | European Union Drug Regulating Authorities Clinical Trials Database (EudraCT) reference: 2020-001904-41  ISRCTN reference: ISRCTN31062548 | | | | | | | | |
|  | 2b | Please see Table 1 | | | | | | | | |
| Protocol version | 3 | Version 4.0 29^th^ September 2020 | | | | | | | | |
| Funding | 4 | The Chief Scientist Office Division (CSO), Scotland. Fujifilm® have are providing Favipirivir free of charge. CRUK have been supportive of core clinical trial unit resource being diverted to support the COVID-19 research effort. | | | | | | | | |
| Roles and responsibilities | 5a | **Dr Janet T Scott**  MRC-University of Glasgow Centre for Virus Research  464 Bearsden Road,  Glasgow, G61 1QH  [Email: janet.scott@glasgow.ac.uk](mailto:janet.scott@glasgow.ac.uk)    **Prof Kevin G Blyth**  University of Glasgow Institute of Cancer Sciences  [Kevin.Blyth@glasgow.ac.uk](mailto:Kevin.Blyth@glasgow.ac.uk)    **Prof Rob Jones**  Beatson West of Scotland Cancer Centre  NHS Greater Glasgow and Clyde  [r.jones@beatson.gla.ac.uk](mailto:r.jones@beatson.gla.ac.uk)    **Prof Emma Thompson**  MRC-University of Glasgow Centre for Virus Research  [Emma.Thomson@glasgow.ac.uk](mailto:Emma.Thomson@glasgow.ac.uk)    **Prof Glenn Burley**  University of Strathclyde  [glenn.burley@strath.ac.uk](mailto:glenn.burley@strath.ac.uk)    **Dr Catherine Hanna**  Beatson West of Scotland Cancer Centre,  NHS Greater Glasgow and Clyde  [Catherine.Hanna@glasgow.ac.uk](mailto:Catherine.Hanna@glasgow.ac.uk)    **Dr Ibrahim Khadra**  University of Strathclyde  [Ibrahim.khadra@strath.ac.uk](mailto:Ibrahim.khadra@strath.ac.uk)    **Samantha Hinsley**  CRUK Clinical Trials Unit  Beatson West of Scotland Cancer Centre  [Samantha.Hinsley@glasgow.ac.uk](mailto:Samantha.Hinsley@glasgow.ac.uk)    **Carol Evans**  Cancer Research UK Clinical Trials Unit  Beatson West of Scotland Cancer Centre  [Email: Carol.Evans@glasgow.ac.uk](mailto:Carol.Evans@glasgow.ac.uk)    **Liz-Anne Lewsley**  Cancer Research UK Clinical Trials Unit  Beatson West of Scotland Cancer Centre  [Email: Liz-Anne.Lewsley@glasgow.ac.uk](mailto:Liz-Anne.Lewsley@glasgow.ac.uk)    **Dr Samantha Carmichael**  Research and Development, NHS Greater Glasgow & Clyde  Ward 11, Dykebar Hospital, Paisley  [Samantha.Carmichael@ggc.scot.nhs.uk](mailto:Samantha.Carmichael@ggc.scot.nhs.uk) | | | | | | | | |
|  |  | **Author contributions**  JS conceived the study. JS, CRH, GB, KB, SC, CE, SH, LAL, IK, RJJ, ET initiated study design and contributed to protocol development. JS is the CSO grant holder. SH provided statistical expertise in trial design. All authors contributed to refinement of the study protocol and approved the final submission to Trials. | | | | | | | | |
|  | 5b | **Trial Co-Sponsor (NHS Greater Glasgow and Clyde) Representative**  Joanne McGarry  Research and Development, NHS Greater Glasgow and Clyde  Ward 11, Dykebar Hospital  Paisley  Tel: 0141 314 4001  [Joanne.McGarry@ggc.scot.nhs.uk](mailto:Joanne.McGarry@ggc.scot.nhs.uk)    **Trial Co-Sponsor (University of Glasgow) Representative**  Debra Stuart  University of Glasgow  Glasgow  Tel: 0141 330 4539  [debra.stuart@glasgow.ac.uk](mailto:debra.stuart@glasgow.ac.uk) | | | | | | | | |
|  | 5c | The main funder (CSO) have no input into study design or analysis. Fujifilm® have had no input into study design but will receive information on severe adverse events and severe unexpected adverse reactions reported during the trial and will be given access to study results at set time-points. The trial sponsors have overall responsibility for the GETAFIX trial and will have representation on the trial management group. The tasks of data management, analysis and publication of results are delegated to the CRUK Clinical Trials Unit Glasgow. | | | | | | | | |
|  | 5d | **Co-ordinating Centre:**  All aspects of trial management and co-ordination including regulatory submissions and data management.  **Membership**  Project Manager: Oversees all aspects from grant application and study development through publication  Clinical Trial Co-ordinator: responsible for site set up and data management  Statistician: involved in study design and analysis of results  Information technology representatives: responsible for MACRO database development and maintenance  Trial monitor: responsible for source data verification.  **Chief investigator and co-investigators**  Design and conduct of GETAFIX  Agreement of final protocol  Preparation of protocol and revisions  Organising steering committee meetings  Publication of study reports  **Trial Steering Group (Chief Investigator, selected co-investigators, project manager, clinical trial co-ordinator, statistician)**  Study planning  Organisation of steering committee meetings  Provide annual risk report MHRA [Medicines and Healthcare Products Regulatory Agency] and ethics committee  SUSAR [Serious unexpected suspected adverse events] reporting  Responsible for trial master file  Budget administration and contractual issues with individual centres  Advice for lead investigators  **Principal investigators and research physicians at individual sites**  All principal investigators will be trial steering group members.  Recruitment of patients and liaising with chief investigator  Reporting of AEs, SAEs, SUSARs  Reviewing progress of study and if necessary agreeing changes to the protocol and/or investigators brochure to facilitate the smooth running of the study. COVID-19 Umbrella Independent Data Monitoring Committee (COVID-19 UIDMC) The role of the COVID-19 UIDMC is to review the accruing trial data and to assess whether they are any safety or efficacy issues that should be brought to participants’ attention or any reasons for the trial not to continue.  In particular they will review the data at the pre-specified interim analyses. The IDMC will be independent of the trial team and will be the only body that may have access to unblinded data during the course of the trial.  It will make recommendations to the UTSC.  **Membership:**  The UIDMC will be chaired by a clinician, with 1 or 2 additional clinical expert (virology, respiratory) members, all with trials experience, and an independent statistician. COVID-19 Umbrella Trial Steering Committee (COVID-19 UTSC) The role of the COVID-19 UTSC is to provide overall supervision of the trial and ensure that it is being conducted in accordance with the principles of GCP and the relevant regulations.  The TSC should agree any significant protocol amendments, provide advice to the investigators on all aspects of the trial and have members who are independent of the investigators, in particular an independent chairperson.  Decisions about continuation or termination of the trial or substantial amendments to the protocol are usually the responsibility of the UTSC.  **Membership:**  The UTSC will be chaired by a clinician, with 1 or 2 additional clinical expert (virology, respiratory) members, all with trials experience, an independent statistician and a patient (PPI) representative. | | | | | | | | |
| **Introduction** |  |  | | | | | | | | |
| Background and rationale | 6a | The new coronavirus (SARS-CoV-2), reported for the first time in China at the end of 2019 belongs to the Coronaviridae family of viruses within the order Nidovirales. Infection with this virus is associated with acute respiratory infection-like symptoms such as pyrexia, cough, chills, and pneumonia. Considering that this viral infection has such a relatively high infectivity and that the outbreak is expected to continue, the development of effective treatments for COVID-19 are urgently needed. A reduction in time to viral clearance could help ameliorate the progression of the disease in severely affected patients and reduce transmission and hospital stay in milder cases.    Favipiravir is an antiviral agent developed by Toyama Chemical Co., Ltd. Favipiravir is approved in Japan for novel or re-emerging influenza virus infection in which other anti-influenza virus agents are not effective or insufficiently effective and the government decides to use the drug as a countermeasure against such influenza viruses. Its mechanism of action is selective inhibition of viral RNA polymerase by its triphosphorylated form (T-705RTP) and it may also be effective for RNA viruses other than influenza virus. It has been reported to be effective against Ebola virus,^1, 2^ Arenaviridae, Lassa Fever, and Bunyaviridae both *in vitro* and *in vivo*.^1,2,3^    Wang *et al*. (2020) reported that the EC_50_ of favipiravir against SARS-CoV-2 virus in Vero E6 cells was 61.88μM.^4^ This corresponds to a concentration of 9.72 μg/ml. By comparison, the EC50 for Ebola zaire was 10.5μg/mL ^4^    [In addition, when favipiravir administered with aerosolized interferon to 36 Chinese patients, shortened the viral clearance time from 11 days to 4, and showed significant improvement in chest imaging (91% vs. 62%) compared to Lopinavir/ritonavir Kaletra®, also administered with aerosolized interferon. Patients taking favipiravir also had fewer adverse events than those taking Lopinavir/ ritonavir 5.](#RANGE!_ENREF_5)    In a clinical pharmacology study in which 1800mg of favipiravir was administered twice daily on Day 1 and 800mg of favipiravir was administered twice daily on Day 2 and thereafter (1800/800mg twice daily) for 22 days to healthy Japanese adult males aged 20 to 39 years, the peak plasma concentration of favipiravir remained at approximately 87 to 104μg/mL on Day 5 and after, and C_min_ remained at approximately 56 to 75μg/mL after the 2nd administration on Day 1. This value exceeds 9.72μg/mL, the EC_50_ of favipiravir for the SARS-CoV-2 virus mentioned above and is significant even when the human protein binding rate of favipiravir of approximately 50% is taken into consideration.    Therefore, we propose that favipiravir administration may achieve exposures in humans that may be potentially efficacious in the treatment of COVID-19. Considering the previous available evidence, the dose proposed for favipiravir for treatment of COVID-19 in the GETAFIX trial is 3600mg on day one followed by 1800mg on days 2 to 10. This should maintain the EC_50_ above the required concentration of 61.88microM (9.72ug/mL) and allow investigation of the efficacy of the single agent regimen. | | | | | | | | |
|  | 6b | Given the rapidly changing landscape with regards to COVID-19 management, the comparator in the GETAFIX trial will be standard care as dictated by the treating physician at the time of study enrolment. | | | | | | | | |
| Objectives | 7 | The working hypothesis for the GETAFIX trial is that favipiravir may be an effective treatment for COVID-19 infection for those patients who have early stage disease, compared to current standard of care. This hypothesis will be tested by evaluating the difference in severity of disease at day 15 as the trial primary endpoint. This study will also provide an important opportunity to evaluate the safety and tolerability of favipiravir, the pharmacokinetic and pharmacodynamic profile of this drug and mechanisms of resistance in the context of COVID-19 infection, the effect of favipiravir on hospitalisation duration, immunological and biometric markers contributing to the clinical condition of patients in the study, and the post COVID-19 health and psycho-social wellbeing of patients recruited to the study. | | | | | | | | |
| Trial design | 8 | The study is designed as an open-label randomised (1:1) phase II/III study which allows for early stopping for lack of efficacy or clear superiority in the experimental arm.  The study is powered to detect an improvement corresponding to an odds ratio of 1.95 in the cumulative odds of the WHO COVID 10-point ordinal severity scale assessed at day 15. This is equivalent to the probability of a patient having a better outcome at this time if allocated to experimental as compared to control of 66% (50% under null hypothesis). The following table illustrates that improvement:- | | | | | | | | |
|  |  |  | | | | | | | | |
|  |  |  | **WHO COVID 10-point ordinal severity scale** | **Assumed percentages on control arm at day 15*** | **Percentages on experimental arm at day 15 with odds ratio=1.95** | |  | | | |
|  |  | Death | Death | 11% | 6.00% | |  |  |  |  |
|  |  | Hospitalized; severe disease | Mechanical ventilation pO2/FIO2<150 and vasopressors, dialysis or ECMO | 5% | 2.90% | |  |  |  |  |
|  |  |  | Mechanical ventilation pO2/FIO2>150 ( SpO2/FIO2> 200) or vasopressors | 7% | 4.40% | |  |  |  |  |
|  |  |  | ICU - intubation & mechanical ventilation, pO2/FIO2>150 or SpO2/FIO2> 200 | 5% | 3.30% | |  |  |  |  |
|  |  | Hospitalized; Mild disease | Hospitalized oxygen by NIV or High flow | 13% | 9.60% | |  |  |  |  |
|  |  |  | Hospitalized on supplemental oxygen | 13% | 11.30% | |  |  |  |  |
|  |  |  | Hospitalized not on supplemental oxygen | 12% | 12.30% | |  |  |  |  |
|  |  | Ambulatory | Symptomatic – assistance needed | 2% | 2.30% | |  |  |  |  |
|  |  |  | Symptomatic – independent | 16% | 21.80% | |  |  |  |  |
|  |  |  | Asymptomatic – viral DNA detected | 16% | 27.00% | |  |  |  |  |
|  |  | *Abstracted from figure 6 in ISARIC COVID-19 Report: 02 April 2020 | | | | | | | | |
|  |  |  | | | | | | | | |
|  |  | To detect this magnitude of difference with 90% power at the 5% 2-sided level of statistical significance requires 302 patients (151 per arm). This allows for the possibility of early stopping at two interim analyses after primary outcome data is available for 128 and 202 patients in total. The interim analyses are detailed in 7.3 of the GETAFIX protocol. | | | | | | | | |
| **Methods: Participants, interventions, and outcomes** | | | | | | | | | | |
| Study setting | 9 | The study population for this trial will include patients aged ≥ 16 years old with a confirmed diagnosis of COVID-19, who are not pregnant or breastfeeding and who have mild symptoms at time of randomisation but who are at high risk of developing severe COVID-19. Patients will be entered into this trial within 96 hours of the COVID19 test being taken. Potentially eligible patients will be identified after community testing, in an acute receiving environment or when admitted to a hospital ward. This study will be conducted in the United Kingdom only. | | | | | | | | |
|  |  | A list of the study sites can be obtained by contacting Mrs Carol Evans at CRUK CTU, Glasgow ([carol.evans@glasgow.ac.uk](mailto:carol.evans@glasgow.ac.uk)). | | | | | | | | |
| Eligibility criteria | 10 | **Inclusion criteria**  1.     Age 16 or over at time of consent  2.     Exhibiting symptoms associated with COVID-19  3.     Positive for SARS-CoV-2 on valid COVID-19 test  4.     Point 1, 2, 3, or 4 on the WHO COVID-19 ordinal severity scale at time of randomisation. (Asymptomatic with positive COVID19 test, Symptomatic Independent, Symptomatic assistance needed, Hospitalized, with no oxygen therapy)  [5.     Have >=10% risk of death should they be admitted to hospital as defined by the ISARIC4C risk index: https://isaric4c.net/risk](file:///C:\Users\cah8z\AppData\Local\Microsoft\Windows\INetCache\Content.Outlook\VNFZKF83\5.    %20Have%20%3e=10%25%20risk%20of%20death%20should%20they%20be%20admitted%20to%20hospital%20as%20defined%20by%20the%20ISARIC4C%20risk%20index:%20https:\isaric4c.net\risk)  6.     Able to provide written informed consent  7.     Negative pregnancy test (women of childbearing potential*)  8.     Able to swallow oral medication    **Exclusion criteria**  1.     Renal impairment requiring dialysis or haemofiltration  2.     Pregnant or breastfeeding  3.     Of child bearing potential* (women), or with female partners of child bearing potential (men) who do not agree to use adequate contraceptive measures for the duration of the study and for 3 months after the completion of study treatment  4.     History of hereditary xanthinuria  5.     Xanthine urinary calculi  6.     Other patients judged unsuitable by the Principal Investigator or sub-Investigator  7.     Known hypersensitivity to favipiravir, its metabolites or any excipients  8.     Severe co-morbidities including patients with severe hepatic impairment defined as:  •       greater than Child-Pugh grade A (Appendix 4)  •       AST or ALT > 5 x ULN  •       AST or ALT > 3x ULN and Total Bilirubin >2xULN  9.     More than 96 hours since first positive COVID19 test was taken.  10.  Unable to discontinue contra-indicated concomitant medications    * Non-childbearing potential must be evidenced by fulfilling one of the following criteria at screening:    Post-menopausal defined as aged more than 50 years and amenorrhoeic for at least 12 months following cessation of all exogenous hormonal treatments.   Irreversible surgical sterilisation by hysterectomy, bilateral oophorectomy or bilateral salpingectomy but not tubal ligation | | | | | | | | |
| Interventions | 11a | Patients who are eligible for this trial will be registered/randomised to receive either:  Control Arm: Standard Treatment for COVID-19 infection  OR  Experimental Arm: Standard Treatment for COVID-19 plus favipiravir:  •       Day 1: Loading Dose favipiravir 1800mg (9 x 200mg tablets) 12 hours apart (2 doses)  •       Days 2 -10: Maintenance Dose favipiravir 800mg (4 x 200mg tablets) every 12hours (18 doses).    *It is strongly advised that the first dose of favipiravir is administered within 24 hours of the treating clinician receiving a positive valid SARS-CoV-2 test result.* | | | | | | | | |
|  | 11b | Subjects entering the study with a Child-Pugh liver impairment of grade A should receive the same loading dose but a reduced maintenance dose of favipiravir 600 mg twice daily on Days 2 through 10.    No dose modifications once on treatment are permitted for toxicity.    A subject should be removed from favipiravir treatment if one of the following criteria is met:  •       AST or ALT > 5 x ULN  •       ALT or AST > 3 x ULN AND total bilirubin > 2X ULN  •       AST or ALT > 3 x ULN AND patient has right upper quadrant pain or eosinophilia  •       Uric acid >20 mg/dL (1.2 mmol/l)  •       Any other ≥ Grade 3 adverse event considered by the study team to be due to the trial drug.    Any > 3 x ULN AST or ALT event should be reported as a AE of Special Interest using the SAE form provided by the CRUK CTU Pharmacovigilance department.    No dose reductions are required for reductions in renal function if they occur during treatment. There are no data for treatment with favipiravir during haemofiltration or dialysis – if this occurs during treatment the Sponsor & CI should be contacted to discuss the risk-benefit of continuing treatment for the individual case.    If remdesivir is administered in combination with favipiravir, EGFR must be >30ml/min and LFTs must be monitored daily during treatment with remdesivir. The stopping rules above for raised transaminases apply for the combination treatment. Ensure remdesivir SmPC or equivalent source of information is consulted before initiating the combination treatment with favipiravir. | | | | | | | | |
|  | 11c | Batch number, dose prescribed, quantity and expiry of the drug supplied will be recorded in the accountability logs. Both bulk stock and individual patient accountability logs will be completed. Favipiravir course will be dispensed for inpatient use in a container and with instructions applied that make it suitable for use at the time of discharge, should continuation be required after discharged to complete the course.    Reasons for any dose delays, dose reductions and dose omissions of trial drugs will be documented in the appropriate section of the CRF and in the patient’s medical records. For patients self-administering favipiravir at home, a diary will be provided to record the time each dose was taken, and the reason for any missed doses – patients will be asked to return the completed diary along with any remaining drug or empty containers at a follow-up clinic visit. | | | | | | | | |
|  | 11d | •       Other medication taken for treatment of COVID-19 whether investigational or approved may be permitted on a case by case basis – please contact Sponsor & CI to discuss.  •       When remdesivir is added to the UK protocols as part of the standard of care for treatment of COVID19, co-prescribing will be permitted with favipiravir with regular monitoring of LFTs according to the combined protocol schedules for both medications. remdesivir prescription must be recorded in the patient CRF.  •       Dexamethasone has been shown to have benefit in the treatment of COVID-19 in certain groups of patients and is being adopted into standard care. Treatment with dexamethasone is permitted concurrently with favipiravir. Co-administration of dexamethasone with favipiravir has not been studied. Dexamethasone is a substrate of CYP3A4 and a moderate inducer of this enzyme. Favipiravir does not appear to be metabolised by this pathway – but there is limited data and Investigators must be vigilant to the possibility of interactions. Dexamethasone prescription must be recorded in the patient CRF.    Details of the administration of any prohibited therapies will be collected on the trial CRFs and must be reported as a protocol deviation as per section 11.3.    There have been limited direct interaction studies of favipiravir and there is limited clinical experience of wide use of the product. Investigators must be vigilant to the possibility of interactions with other medicines other than those listed below.  •       Paracetamol AUC may be increased and dosage should be restricted to 3g in 24h – this may require to be lowered further for lower bodyweight patients. This should be highlighted on the patient’s notes, and clear instruction given to patients treated in the community.    The following may interact with favipiravir and risks and benefits should be carefully considered prior to treatment with the following:  •       Any other anti-viral medication whether investigational or approved.  •       Any drugs known to significantly inhibit Aldehyde Oxidase activity (e.g., pyrazinamide, amitriptyline, chlorpromazine, clomipramine, clozapine, erythromycin, ketoconazole, nortriptyline, quetiapine, raloxifene, perphenazine, promethazine, propafenone, tamoxifen, thioridazine).  Any drugs metabolized by the Aldehyde Oxidase pathway (e.g., famciclovir, hydralazine, lamivudine, sulindac, zaleplon, ziprasidone).    •       Any drugs that interact with xanthine oxidase pathway, e.g. theophylline and aminophylline.  •       Any drugs metabolised by CYP2C8, e.g. concentration of repaglinide concentration may be increased.  •       Drugs with possible drug-drug interactions (concomitant medications requiring particular attention – see the Investigator Brochure).    This list is not exhaustive and IB should be checked in addition. | | | | | | | | |
| Outcomes | 12 | Primary, secondary, and other outcomes, including the specific measurement variable (eg, systolic blood pressure), analysis metric (eg, change from baseline, final value, time to event), method of aggregation (eg, median, proportion), and time point for each outcome. Explanation of the clinical relevance of chosen efficacy and harm outcomes is strongly recommended | | | | | | | | |
| Participant timeline | 13 | Please see Figure 1 | | | | | | | | |
| Sample size | 14 | The study is powered to detect an improvement corresponding to an odds ratio of 1.95 in the cumulative odds of the WHO COVID 10-point ordinal severity scale assessed at day 15. This is equivalent to the probability of a patient having a better outcome at this time if allocated to experimental as compared to control of 66% (50% under null hypothesis).    To detect this magnitude of difference with 90% power at the 5% 2-sided level of statistical significance requires 302 patients (151 per arm). | | | | | | | | |
| Recruitment | 15 | Potential participants may be identified before their COVID-19 test result is available and will be randomised within 96 hours of their COVID-19 test being taken. They may be identified either by clinical staff involved in their treatment as per standard of care, or by a clinical member of the GETAFIX trial team. COVID-19 testing centres may provide adverts to attendees who may contact the GETAFIX trial team if interested in participating. Following this, patients will be contacted by a clinical member of the GETAFIX trial team who will explain to the patient that they may be potentially eligible for a clinical trial. If the patient is interested in hearing more about the trial, this option will be discussed further with the patient. | | | | | | | | |
| **Methods: Assignment of interventions (for controlled trials)** | | | | | | | | | | |
| Allocation: |  |  | | | | | | | | |
| Sequence generation | 16a | Computer-generated random numbers will be used for allocation sequence generation. A minimisation algorithm incorporating a random component will be used to allocate patients to either experimental or control arm. The factors used in the minimisation will be:  •       Site  •       Age (16 - 50; 51 – 70; 71+)  •       History of hypertension or currently obese (BMI>30 or obesity clinically evident) (yes; no)  •       <7 days duration of symptoms (yes; no; unknown)  •       Sex (male; female).  •       WHO COVID ordinal severity score at baseline (1; 2 or 3; 4) | | | | | | | | |
| Allocation concealment mechanism | 16b | After the patient has provided informed consent to participate in the trial, the CRUK CTU, Glasgow will provide the site with a screening number for the patient, and screening may commence. No screening activities related to the trial may be undertaken until informed consent has been obtained. Once the patient has been deemed eligible for the trial by the PI or medically trained designee, 1:1 randomisation into the Control Arm or the Experimental Arm will occur. In order for randomisation to occur, the participating site must fax, telephone or email the CRUK Clinical Trials Unit, Glasgow. The PI or medically trained designee will have no access to or control over the allocation sequence generator which will be done centrally. | | | | | | | | |
| Implementation | 16c | Allocation sequence generation will be performed by the CRUK Clinical Trials Unit in Glasgow. | | | | | | | | |
| Blinding (masking) | 17a | Neither patients, nor the treating clinician will be blinded to treatment allocation. | | | | | | | | |
|  | 17b | Not applicable | | | | | | | | |
| **Methods: Data collection, management, and analysis** | | | | | | | | | | |
| Data collection methods | 18a | The WHO COVID-19 severity scale will be used for the primary endpoint assessment. | | | | | | | | |
|  | 18b | In many circumstances where a patient withdraws from trial treatment, it will, nonetheless, be desirable for the patient to remain on the trial (for example for follow-up purposes). If, however, the patient withdraws from the trial itself, it should be clearly documented in the patient’s notes what they are withdrawing from (consent to use any past data, consent to use any samples collected or consent for further data collection from the date of consent withdrawal). If a patient withdraws their consent from the trial, the site must contact the CRUK CTU Glasgow with full details of the withdrawal. Where applicable, the CRUK CTU Glasgow may ask the site to complete a Consent Withdrawal Form to record full details of the consent withdrawal. In the event of withdrawal, data or tissue already collected with consent will be retained and used in the study, but no further tissue will be collected or any other research procedures carried out on or in relation to the participant. | | | | | | | | |
| Data management | 19 | The CRFs for this trial will be completed using the electronic remote data capture (eRDC) system, MACRO. CRUK CTU will regularly review the data for compliance with the protocol, and for inconsistent or missing data.  Should any missing data or data anomalies be found within the eCRFs upon CTU review, queries will be generated within the MACRO^®^ study database for the site to access and resolve. E-Forms will also have inbuilt validations to generate autoqueries when data incomplete or inconsistent. Details are in protocol. | | | | | | | | |
| Statistical methods | 20a | Analyses to account for any non-trial treatment for COVID-19 will be investigated prior to any analyses being undertaken. The decision regarding whether adjustments are required may be based on a blinded assessment of the number of patients who receive non-trial treatment for COVID19 prior to each outcome time-point being reached. Analyses will be used to remove confounding from non-trial COVID19 treatment, and may include inverse probability weighting.    The primary analysis will use cumulative odds ordinal regression with proportional odds. The model terms will be study arm and the minimisation factors used for randomisation. The odds of a patient having a better outcome if allocated to the experimental arm will be estimated along with the associated 95% confidence interval. A Forest plot illustrating treatment effect odds ratios by minimisation factor categories will also be provided. . A test for interaction will be conducted to assess whether the effect of treatment arm depends on the other clinical factors used in the minimisation algorithm.      It will not be possible to distinguish between 0 and 1 on the WHO COVID 10-point ordinal severity scale so these will be combined in analyses. | | | | | | | | |
|  | 20b | Proportion of patients meeting level 7 or above on the WHO COVID 10 point ordinal severity scale or dead by day 29; Overall survival over the 60 days; Viral clearance on or before d8 of treatment (inpatients)    These end-points will be tabulated and examined using logistic regression incorporating terms for study arm and the minimisation factors used for randomisation.    Duration of pyrexia  This will be summarised as time from developing pyrexia (taken as the date of admission to hospital if present at randomisation) using a Cumulative Incidence Function plot and analysed using Fine and Gray regression incorporating terms for study arm and the minimisation factors used for randomisation. (Gray, 1988, a class of k-sample tests for comparing the cumulative incidence of a competing risk. Annals of Statistics 1988; 16:1141-1154)(Fine, 1999, A proportional hazards model for the sub distribution of a competing risk. Journal of the American Statistical Association 1999; 94: 496-509). Patients who die during the course of the 60 day study period, prior to resolution of pyrexia, will be considered as having had a competing risk at the date of death. Participants who never develop pyrexia will not be included in this analysis.    WHO COVID 10-point ordinal severity scale at day 8, 29, 60    This will be analysed using the same technique as the primary end-point.    Safety    The worst toxicity grades (including AEs of special interest) experienced during active treatment will be compared between the study arms using the Mann-Whitney U test. P-values will be interpreted in the context of the multiple tests performed. This analysis with be restricted to events that occur in more than 10% of patients in either arm. All Serious Adverse Events (including SARs and SUSARs) will be tabulated.    Details of the approach for analysis of exploratory endpoints are detailed in the GETAFIX trial protocol. | | | | | | | | |
|  | 20c | All analyses will be performed on the ITT population (all randomised patients) or safety population (all patients, with experimental arm patients not receiving at least one dose of favipiravir analysed as part of the control arm).  Methods required to handle missing data, such as multiple imputation, will be defined in the statistical analysis plan. | | | | | | | | |
| **Methods: Monitoring** | | | | | | | | | | |
| Data monitoring | 21a | The role of the COVID-19 UIDMC is to review the accruing trial data and to assess whether they are any safety or efficacy issues that should be brought to participants’ attention or any reasons for the trial not to continue. In particular they will review the data at the pre-specified interim analyses. The IDMC will be independent of both the investigators and will be the only body that may have access to unblinded data during the course of the trial. It will make recommendations to the trial steering committee.    The Clinical Trial Project Manager will be responsible for the ongoing coordination and management of the trial. The Sponsors are responsible for all duties relating to pharmacovigilance in accordance with section 7. Before the trial can be initiated, the prerequisites for conducting the trial must be clarified and the organisational preparations made with the trial centre. The Sponsor must be informed immediately of any change in the personnel involved in the conduct of the trial.  During the trial, the Sponsor’s Clinical Trial Monitor (CTM) is responsible for monitoring data quality in accordance with Sponsor’s standard operating procedures (SOPs).    Development Safety Update Reports (DSURs) will be prepared for the trial. The reports will be written by the CI and CRUK CTU on behalf of the Sponsor. The CRUK CTU will submit DSURs to the Regulatory Authorities, REC, trial sites, Sponsor and Fuji Film Pharmaceuticals who are supplying the IMP on the anniversary of obtaining the UK Clinical Trial Authorisation.    Participating study sites will be monitored remotely by the CRUK CTU on behalf of the Sponsor(s) by both telephone and remote review of data. The CRUK CTU reserves the right to undertake for-cause monitoring should this be considered necessary at any stage. The level of monitoring have been agreed in advance between the Project Manager and Clinical Trial Monitor with input from the Quality Assurance Manager and Sponsor via the trial risk assessment. The PI and Site Pharmacy staff will allow the Clinical Trial Monitor remote access to relevant trial data as requested. Investigators and site staff will be notified in advance about forthcoming monitoring. | | | | | | | | |
|  | 21b | The stopping rules for the interim analyses are based on group sequential asymmetric boundaries using Hwang-Shih-DeCani spending functions with gamma=-2.6 (lower boundary) and gamma=-9.5 (upper boundary). The upper boundary is binding for early stopping; the lower boundary is non-binding and the Data Monitoring Committee may take into account the effect on secondary end-points in reaching their decision on whether the study should proceed or not. | | | | | | | | |
|  |  |  | | | | | | | | |
|  |  |  |  | Lower boundary | | Upper boundary | | | | |
|  |  | Analysis | N | Z | Nominal p | | Beta-Spend | Z | Nominal p | Alpha -Spend |
|  |  | Interim 1 | 128 | 0 | 0.502 | | 0.016 | 3.72 | 0.0001 | 0.0001 |
|  |  | Interim 2 | 202 | 0.84 | 0.7992 | | 0.0218 | 3.08 | 0.001 | 0.001 |
|  |  | Final | 302 | 1.96 | 0.9751 | | 0.0622 | 1.96 | 0.0249 | 0.0239 |
|  |  | *Total* |  |  |  | | *0.1* |  |  | *0.025* |
|  |  |  | | | | | | | | |
|  |  |  | | | | | | | | |
|  |  | At the first interim analyses the distribution of the primary end-point in the control arm will be reviewed by the Trials Steering Committee (TSC) to determine how well it conforms to the initial assumptions and whether or not any modifications may be required to the sample size calculations. This review is required because of limited experience with the primary end-point in this patient population. The TSC has no access to outcome data on the experimental arm. | | | | | | | | |
| Harms | 22 | Safety reporting will be performed by the Pharmacovigilance Department of the CRUK CTU Glasgow as delegated by the trial Sponsor.    Participants will be asked at each trial visit about the occurrence of AEs since their last visit.  AEs will be recorded, notified, assessed, reported, analysed and managed in accordance with the Medicines for Human Use (Clinical Trials) Regulations 2004 (as amended) and the trial protocol. Full details about the definitions and reporting of AEs, SAEs and SUSARs, and the actions that will be taken in response to these events can be found in the GETAFIX protocol. | | | | | | | | |
| Auditing | 23 | CRUK CTU will regularly review the data for compliance with the protocol, and for inconsistent or missing data. Should any missing data or data anomalies be found within the eCRFs upon CTU review, queries will be generated within the MACRO^®^ study database for the site to access and resolve. Sites are expected to review and respond to queries within the database in a timely manner (within 2-3 weeks). Any issues identified at sites in relation to poor data/slow response to data queries will be managed as per the data escalation process below. | | | | | | | | |
| **Ethics and dissemination** | | | | | | | | | | |
| Research ethics approval | 24 | This trial has revived approval from the West of Scotland Research Ethics Committee on 20^th^ May 2020 (REC reference: 20/WS/0073). | | | | | | | | |
|  |  | Any change to the trial protocol will require an amendment. Any proposed, non-administrative, protocol amendments will be initiated by the CI following discussion with the TMG and any required amendment forms will be submitted to the regulatory authority, ethics committee and sponsor(s). The CI and the TMG will liaise with trial sponsor(s) to determine whether an amendment is non-substantial or substantial. All amended versions of the protocol will be signed by the CI and sponsor(s) representative. Before the amended protocol can be implemented favourable approval must be sought from the original reviewing REC, trial Sponsors, MHRA and participating site R&D offices.  Important protocol modifications will reported by email to ISCTRN and to all principal investigators. Trial participants will be made aware of any substantial protocol amendments by the principal investigator or designee at site. | | | | | | | | |
| Consent or assent | 26a | After receiving verbal and written information, patients will be given sufficient time to decide whether or not they would like to take part in the trial. All efforts will be made to ensure that patients understand the commitment required to fulfil the trial requirements and facilitate discussion with friends and relatives at the request of the patient. Patients will be told that their participation is entirely voluntary and that they can leave the trial at any time without their standard care being affected. Patients will then be given the choice of whether they wish to participate. Signed participant consent must be obtained. For patients who have capacity to understand and give consent, but are unable to read the consent form or sign their name, witness consent may be given. The consent forms should also be signed by the person undertaking the consent procedure at site, who must be detailed on the delegation log as having this authorisation. The Principal Investigator is responsible for ensuring the designee is suitably qualified by training or experience to take informed consent, if consent is delegated to a designee.    Patients who become unwell to such an extent that they lose capacity during the course of the study will be permitted to continue on IMP until the point they are unable to swallow oral medication, and all other study processes, including safety assessments, should continue. No patient whether they have capacity or not will continue on the trial if it is unsafe to do so in the opinion of the Principal or Chief Investigator, or the Trial Steering Committee. | | | | | | | | |
|  | 26b | There will be an optional consent form for patients who may want to contribute to more frequent PK and PD sampling. The maximum number of patients who will undergo this testing will be sixteen, eight males and eight females. This option will be offered to all patients who are being treated in hospital at the time of taking informed consent, however only patients in the experimental arm of the trial will be able to undergo this testing. | | | | | | | | |
| Confidentiality | 27 | All information collected during the course of the trial will be kept strictly confidential.  Information will be held securely on paper and electronically at the CRUK CTU. The CRUK CTU will comply with all aspects of the 1998 Data Protection Act and operationally this will include:  •       Consent from participants to record personal details including initials, date of birth, CHI/NHS number, hospital number, GP name and address.  •       Appropriate storage, restricted access and disposal arrangements for patient’s personal and clinical details  •       Consent from participants for access to their medical records by responsible individuals from the research staff or from regulatory authorities, where it is relevant to trial participation  •       Consent from participants for the data collected for the trial to be used to evaluate safety and develop new research.  •       Where central monitoring of source documents by CRUK CTU (or copies of source documents) are required (such as scans or local blood results), the patient’s name must be obliterated by site before sending.  •       Where anonymisation of documentation is required, sites are responsible for ensuring only the instructed identifiers are present before sending to CRUK CTU.    If a participant withdraws consent from further trial treatment and / or further collection of data their samples will remain on file and will be included in the final trial analysis unless they specifically withdraw consent for this. | | | | | | | | |
| Declaration of interests | 28 | The authors listed on this protocol have no competing interests to declare. | | | | | | | | |
| Access to data | 29 | The data arising from GETAFIX will belong to the trial Sponsors (NHS Greater Glasgow and Clyde and University of Glasgow). The TMG shall act as custodian of this data. | | | | | | | | |
| Ancillary and post-trial care | 30 | No special insurance is in place for patients in this trial other than standard NHS liability insurance  providing indemnity against clinical negligence. This does not provide cover for non-negligence e.g.  harm caused by an unexpected side effect of participating in a trial. The sponsors have responsibility  for ensuring that financial cover for damages or compensation arising from no fault harm is available  to patients, where applicable. The co-sponsor, University of Glasgow, maintains clinical trials  insurance. Cover for this clinical trial has been agreed under the current policy. | | | | | | | | |
| Dissemination policy | 31a | The GETAFIX TMG is responsible for approving the content and dissemination of all publications, abstracts and presentations arising from the trial and for assuring the confidentiality and integrity of the trial. | | | | | | | | |
|  | 31b | It will provide collaborators the International Committee of Medical Journal Editors (ICMJE)(http://www.icmje.org/recommendations/browse/roles-and-responsibilities/defining-the-role-of-authors-and-contributors.html#two) used to ensure all those who have contributed to the study are appropriately acknowledged.  No site or individual will publish data without prior approval of the TMG. | | | | | | | | |
|  | 31c | The protocol will be available via this Letter to the Editor and the main protocol details are publicly available with the GETAFIX ISRCTN record. There are currently no plans to grant public access to the participant-level dataset or the statistical code | | | | | | | | |
| **Appendices** |  |  | | | | | | | | |
| Informed consent materials | 32 | A trial patient information sheet will be given to participants and authorised surrogates. | | | | | | | | |
| Biological specimens | 33 | The Pharmacokinetic and Pharmacodynamic specimen collection schedule is detailed in an appendix of the GETAFIX protocol. The GETAFIX laboratory manual contains details of biological specimen collection. | | | | | | | | |
|  |  | Pharmacokinetic and Pharmacodynamic analyses will be performed by The Pharmacy department, University of Strathclyde, and the University of Glasgow Translational Pharmacology Laboratory respectively. | | | | | | | | |

Table 1

| **Data category** | Information |
| --- | --- |
| **Primary Registry and Trial Identifying Number** | European Union Drug Regulating Authorities Clinical Trials (EudraCT) Database; Reference number 2020-001904-41. |
| **Date of Registration in Primary Registry** | 15th April 2020 |
| **Secondary Identifying Numbers** | ISRCTN31062548. (Registered 7th September 2020) |
| **Source(s) of Monetary or Material Support** | Chief Scientist Office Division Scotland, Cancer Research UK, FUJIFILM Toyama Chemical Co |
| **Primary Sponsor** | University of Glasgow and NHS Greater Glagsow and Clyde (joint primary sponsor) |
| **Secondary Sponsor(s)** | NA |
| **Contact for Public Queries** | [carol.evans@glasgow.ac.uk](mailto:carol.evans@glasgow.ac.uk) |
| **Public Title** | GETAFIX (Glasgow Early Treatment Arm Favipiravir) – a study to compare the effectiveness of adding the antiviral drug favipiravir to standard care in COVID-19 patients, compared with standard care alone. |
| **Scientific Title** | Glasgow Early Treatment Arm Favipiravir : A randomised controlled study of favipiravir as an early treatment arm in COVID-19 patients |
| **Countries of Recruitment** | United Kingdom |
| **Health Condition(s) or Problem(s) Studied** | Patients with SARS-CoV-2 infection (COVID-19). |
| **Intervention(s)** | Favipiravir plus standard care versus standard care alone |
| **Key Inclusion and Exclusion Criteria** | Inclusion: 1. Age 16 or over at time of consent 2. Exhibiting symptoms associated with COVID-19 3. Positive for SARS-CoV-2 on valid COVID-19 test 4. Point 1, 2, 3, or 4 on the WHO COVID-19 ordinal severity scale at time of randomisation. (Asymptomatic with positive valid COVID19 test, Symptomatic Independent, Symptomatic assistance needed, Hospitalized, with no oxygen therapy) 5. Have >=10% risk of death should they be admitted to hospital as defined by the ISARIC4C risk index: https://isaric4c.net/risk 6. Able to provide written informed consent 7. Negative pregnancy test (women of childbearing potential*) 8. Able to swallow oral medication |
|  | Exclusion 1. Renal impairment requiring, or likely to require, dialysis or haemofiltration 2. Pregnant or breastfeeding 3. Of child bearing potential* (women), or with female partners of child bearing potential (men) who do not agree to use adequate contraceptive measures for the duration of the study and for 3 months after the completion of study treatment 4. History of hereditary xanthinuria 5. Other patients judged unsuitable by the Principal Investigator or sub-Investigator 6. Known hypersensitivity to favipiravir, its metabolites or any excipients 7. Severe co-morbidities including: patients with severe hepatic impairment, defined as: • (greater than Child-Pugh grade A- see Appendix 4, • AST or ALT > 5 x ULN • AST or ALT >3 x ULN and Total Bilirubin > 2xULN 8. More than 96 hours since first positive COVID19 test sample was taken 9. Unable to discontinue contra-indicated concomitant medications (section 6.7) |
| **Study Type** | Open label randomised phase II/III superiority trial |
| **Date of First Enrollment** | TBC |
| **Sample Size** | Planned sample size n=302 |
| **Recruitment Status** | Recruiting |
| **Primary Outcome(s)** | Efficacy of favipiravir in addition to standard care in patients with COVID-19 in reducing the severity of disease compared to standard care alone |
| **Key Secondary Outcomes** | Proportion of patients meeting level 7 or above on the WHO COVID 10 point ordinal severity scale or dead by day 29; WHO COVID 10 point ordinal severity scale level days 8/29/60; Overall survival; Viral clearance on or before d8 (inpatients only); Duration of pyrexia in days (defined as a temperature ≥ 38 degrees Celcius) (inpatients only) |
| **Ethics Review** | Approval by West of Scotland Research Ethics Service May 2020 |
| **Completion date** | Not yet available |
| **Summary Results** | Not yet available |
| **IPD sharing statement** | The Clinical Trials Unit (Glasgow), which is co-ordinating the trial, will collect the initials, date of birth and NHS number or Community Health Index (CHI) number for patients at the time they are registered on to the trial. This information will be stored securely and will be kept strictly confidential, with access provided only to authorised personnel.  NHS Greater Glasgow &Clyde and the University of Glasgow are the sponsors for this study based in the United Kingdom, will be using information from patients and their medical records in order to undertake this study and will act as the data controller for this study. The Co-Sponsor will keep identifiable information about patients for 10 years after the study has finished.  Further information about how we use patient information can be found at: <https://www.hra.nhs.uk/information-about-patients/> |

**Table 2a: Schedule of Events for inpatients**

| Investigation | Screening | Day 1 | Day 2 | Day 3 | Day 4 | Day 5 | Day 6 | Day 7 | | Day 8 | Day 9 | Day 10 | Day 15^5^ | Follow-up(day 29 )^6^ | Follow-up (day 60 )^6^ | |
| --- | --- | --- | --- | --- | --- | --- | --- | --- | --- | --- | --- | --- | --- | --- | --- | --- |
| Written informed consent | X |  |  |  |  |  |  |  | |  |  |  |  |  |  | |
| Demographic and Medical History | X |  |  |  |  |  |  |  | |  |  |  |  |  |  | |
| Randomisation | X |  |  |  |  |  |  |  | |  |  |  |  |  |  | |
| Observations^1^ | X | X | X | X | X | X | X | X | | X | X | X | X | X | X | |
| Height and weight | X |  |  |  |  |  |  |  | |  |  |  | X | X | X | |
| Physical Exam | X | Symptom directed where clinically indicated | | | | | | | | | | | | | | |
| AE Evaluation | Continually review from time of informed consent | | | | | | | | | | | | | | | |
| Concomitant Medications | Continually review from time of informed consent | | | | | | | | | | | | | | | |
| IMP Dispensing |  | X |  |  |  |  |  | |  |  |  |  |  |  |  |  |
| IMP Administration^2^ |  | X | X | X | X | X | X | | X | X | X | X |  |  |  |  |
| WHO performance status  (Appendix 2) | X |  |  |  |  |  |  | |  |  |  |  |  |  |  |  |
| WHO COVID Ordinal Severity Scale(Appendix 1) | X | X | X | X | X | X | X | | X | X | X | X | X | X | X |  |
| Routine Clinical Blood tests^3^  (see table 2) | X | X |  | X |  |  |  | |  | X |  |  | X | X | X |  |
| Blood Samples for Epigenetic and Immunological testing^3^ |  | X^10^ |  | X^10^ |  |  |  | |  |  |  |  | X | X | X |  |
| Viral PCR (nasopharyngeal swab)^3^ | X^8^ | X^4^ |  | X |  |  |  | |  | X |  | X | X | X | X |  |
| Viral Load (nasopharyngeal swab)^3^ |  | X^4^ |  | X |  |  |  | |  | X |  | X | X | X | X |  |
| Pregnancy Test^7^ | X |  |  |  |  |  |  | |  |  |  |  |  |  |  |  |
| PK testing^9^ |  | X |  | X |  | X |  | |  | X |  | X |  |  |  |  |
| COVID19 Health and Wellbeing Follow up Survey (Appendix 3) |  |  |  |  |  |  |  | |  |  |  |  | X | X | X |  |

1. Observations: Heart rate, temperature, oxygen saturations, blood pressure, respiratory rate
2. IMP should start to be administered within 24 hours of randomisation
3. Bloods/Nasopharyngeal swabs can be taken +/- 48 hours from scheduled time point unless otherwise specified.
4. Nasopharyngeal swabs on day 1 should be taken as close to T0 (time of first drug administration) as possible
5. Patients discharged prior to day 15 should follow the outpatient schedule of assessments from point of discharge. Patients should return for clinic visit where possible, or be followed up by phone call if unable to attend
6. Follow-up visit at day 29 and 60 may be performed by telephone if patient is unable to attend clinic
7. Serum pregnancy test, for patients of child bearing potential only
8. Screening COVID-19 test can be done using any valid method of testing
9. PK Samples should be taken at the following times:

- Day 1: pre dose and 0.5 hours post dose
- Day 3: pre and 0.5 hours post dose
- Day 5: pre and0.5 hours post dose
- Day 8: Pre dose
- Day 10: Pre dose

In addition, 16 volunteers (8 male, 8 female) will have more frequent sampling for PK on days 1 and 3 after either dose that day. Hours post dose: 0.5, 1, 2, 7, 12

**PK samples require to be spun within 4 hours of being taken – where sites do not have the facility to do this, PK’s will be omitted.**

1. Blood samples for epigenetic and immunological testing should be taken at the time of the first PK draws on days 1 and 3 (i.e. pre-dose)

**Table 2b: Schedule of Events for outpatients**

| Investigation | Screening | Day 1 | Day 2 | Day 3 | Day 4 | Day 5 | Day 6 | Day 7 | Day 8 | Day 9 | Day 10 | Day 15 | Follow-up(day 29)^5^ | Follow-up(day 60)^5^ |
| --- | --- | --- | --- | --- | --- | --- | --- | --- | --- | --- | --- | --- | --- | --- |
| Written informed consent | X |  |  |  |  |  |  |  |  |  |  |  |  |  |
| Demographic and Medical History | X |  |  |  |  |  |  |  |  |  |  |  |  |  |
| Randomisation | X |  |  |  |  |  |  |  |  |  |  |  |  |  |
| Observations^1^ | X |  |  |  |  |  |  |  |  |  |  | X | X | X |
| Height and weight | X |  |  |  |  |  |  |  |  |  |  | X | X | X |
| Physical Exam | X |  |  |  |  |  |  |  |  |  |  | Symptom directed where clinically indicated | | |
| AE Evaluation | X | Continually review from time of consent ^8^ | | | | | | | | | | | | |
| Concomitant Medications | X | Continually review from time of consent^8^ | | | | | | | | | | | | |
| IMP Dispensing^10^ |  | X |  |  |  |  |  |  |  |  |  |  |  |  |
| IMP Administration^2^ |  | X | X | X | X | X | X | X | X | X | X |  |  |  |
| WHO performance status (Appendix 2) | X |  |  |  |  |  |  |  |  |  |  |  |  |  |
| WHO COVID Ordinal Severity Scale  (Appendix 1)^8^ | X | X | X | X | X | X | X | X | X | X | X | X | X | X |
| Routine Clinical Blood tests^3^ (see table 2) | X |  |  |  |  |  |  |  |  |  |  | X | X | X |
| Blood Sample for PK analysis^11^ (favipiravir arm only) | X^11^ | X^11^ |  |  |  |  |  |  |  |  |  |  |  |  |
| Blood Samples for Epigenetic and Immunological testing^3^ | X |  |  |  |  |  |  |  |  |  |  | X | X | X |
| Viral PCR (nasopharyngeal swab)^3^ | X^7^ |  |  |  |  |  |  |  | X^9^ |  |  | X | X | X |
| Viral Load (nasopharyngeal swab)^3^ | X |  |  |  |  |  |  |  | X^9^ |  |  | X | X | X |
| Pregnancy Test^6^ | X |  |  |  |  |  |  |  |  |  |  |  |  |  |
| COVID19 Health and Wellbeing Follow up Survey (Appendix 3) |  |  |  |  |  |  |  |  |  |  |  | X | X | X |

1. Observations: Heart rate, temperature, oxygen saturations, blood pressure, respiratory rate
2. IMP should start to be administered within 24 hours of randomisation
3. Bloods/Nasopharyngeal swabs can be taken +/- 48 hours from scheduled time point unless otherwise specified
4. Nasopharyngeal swabs on day 1 should be taken as close to T0 (time of first drug administration) as possible
5. 5.Follow-up visit at day 15, 29 and may be performed by telephone if patient is unable to attend clinic
6. Serum pregnancy test, for patients of childbearing potential only. Result must be confirmed negative before IMP is released to patient.
7. Screening COVID-19 test can be done using any valid method of testing
8. AE, con med and WHO COVID Ordinal Severity Scale assessments should be made by phone call on days 1 – 10.
9. Optional nasopharyngeal swab may be done by patient at home if possible and safe to transport
10. To minimise the time spent in the clinic, patients can return home after screening and have IMP delivered to their home following randomisation
11. PK sampling should only be done where site have provision to take a predose sample (screening or day 1 pre-dose) AND a post dose sample (30 minutes after dosing), and have facilities to spin samples within 4 hours.
